# Defining the functional traits that drive bacterial decomposer community productivity

**DOI:** 10.1038/ismej.2017.22

**Published:** 2017-03-21

**Authors:** Rachael Evans, Anna M Alessi, Susannah Bird, Simon J McQueen-Mason, Neil C Bruce, Michael A Brockhurst

**Affiliations:** 1 Department of Biology, University of York, York, UK; 2 Department of Animal and Plant Sciences, University of Sheffield, Sheffield, UK

## Abstract

Microbial communities are essential to a wide range of ecologically and industrially important processes. To control or predict how these communities function, we require a better understanding of the factors which influence microbial community productivity. Here, we combine functional resource use assays with a biodiversity–ecosystem functioning (BEF) experiment to determine whether the functional traits of constituent species can be used to predict community productivity. We quantified the abilities of 12 bacterial species to metabolise components of lignocellulose and then assembled these species into communities of varying diversity and composition to measure their productivity growing on lignocellulose, a complex natural substrate. A positive relationship between diversity and community productivity was caused by a selection effect whereby more diverse communities were more likely to contain two species that significantly improved community productivity. Analysis of functional traits revealed that the observed selection effect was primarily driven by the abilities of these species to degrade β-glucan. Our results indicate that by identifying the key functional traits underlying microbial community productivity we could improve industrial bioprocessing of complex natural substrates.

## Introduction

Microbial communities underpin the functioning of natural ecosystems (Soliveres *et al.*, 2016) and the efficiency of a wide range of industrial bioprocesses (for example, waste bioreactors) (Cydzik-Kwiatkowska and Zielińska, 2016; Widder *et al.*, 2016). The form of the biodiversity–ecosystem functioning (BEF) relationship is therefore an important property of microbial communities both in nature and the simpler communities used in a range of industrial bioprocesses. Several studies have identified positive BEF relationships for microbial community productivity (Bell *et al.*, 2005; Gravel *et al.*, 2011), stability (Awasthi *et al.*, 2014), micropollutant degradation (Johnson *et al.*, 2015) and resistance to invasion (Elsas *et al.*, 2012), suggesting that for a range of functions microbial community performance improves with increasing species richness. Positive BEF relationships can arise via the complementarity effect, whereby diverse communities use more of the available resource space through niche differentiation or facilitation (Salles *et al.*, 2009; Singh *et al.*, 2015), or the selection effect (also termed the sampling effect), whereby diverse communities are more likely to contain species which have a large impact on community functioning (Hooper *et al.*, 2005; Langenheder *et al.*, 2012, 2010; Awasthi *et al.*, 2014). Both complementarity and selection effects depend on the functional traits of constituent species and several studies have now shown functional diversity to be a better predictor of community function than phylogenetic diversity (Mokany *et al.*, 2008; Salles *et al.*, 2009; Krause *et al.*, 2014). However, for many ecologically and biotechnologically important microbial communities it is still unclear how the functional traits of individual species scale-up to determine the performance of a diverse community.

One of the most important ecosystem functions microbial communities perform is the decomposition of plant material and subsequent nutrient cycling (Van Der Heijden *et al.*, 2008; McGuire and Treseder, 2010). Understanding the decomposition of plant material also has important industrial relevance. Plant biomass (collectively referred to as lignocellulose) is the most abundant raw material on Earth (Pauly and Keegstra, 2008). It is typically composed of approximately 40–50% cellulose, 20–40% hemicellulose and 20–35% lignin which together form a complex, recalcitrant structure (Himmel *et al.*, 2007; Liao *et al.*, 2016). The high sugar content and abundance of lignocellulose make it a promising substrate for biofuel production (Naik *et al.*, 2010). However, lignin is highly recalcitrant to enzymatic attack causing a bottleneck in the efficient conversion of lignocellulose to biofuels reducing cost-effectiveness (Jorgensen *et al.*, 2007; Naik *et al.*, 2010). Understanding how natural microbial communities (for example, in soils (Lynd *et al.*, 2002), compost (Lopez-Gonzalez *et al.*, 2014) or termite guts (Brune, 2014)) achieve efficient lignocellulose degradation could inform both the prediction of nutrient cycling in natural systems and the design of efficient microbial communities for industrial processes (Wei *et al.*, 2012). Both biodiversity and the presence of certain species have been shown to influence the rate of decomposition by bacterial communities (Bell *et al.*, 2005; Bonkowski and Roy, 2005; Langenheder *et al.*, 2012) but the mechanisms which determine community decomposition performance remain poorly understood (McGuire and Treseder, 2010). A key question therefore is to what extent community functioning is predictable from the combined functional traits of constituent species?

Using culturable bacterial strains isolated from compost we performed a random partition design BEF experiment (Bell *et al.*, 2009) to test the contributions of species richness and composition to productivity of communities when grown on wheat straw. Although using only the culturable fraction of the community is likely to overlook some functionally important species in the natural community, culturability is a key feature of microbes that could feasibly be used in industrial bioprocessing. Next we tested how the functional traits of individual species shaped the productivity of these communities to determine the extent to which community productivity was predictable from the functional traits of the constituent species and to determine the contribution of each functional trait to overall productivity. We quantified the functional resource use traits of each species by their ability to utilise a range of known components of lignocellulose (that is, cellulose, hemicellulose, pectin and lignin).

## Materials and methods

### Bacterial isolates

Bacterial strains used in this study were isolated from wheat straw compost enrichment cultures (700 ml M9 minimal media (22 mm KH_2_PO_4_, 42 mm Na_2_HPO_4_, 19 mm NH_4_Cl, 1 mm MgSO_4_, 0.09 mm CaCl_2_, 9 mm NaCl), 1% (w/v) wheat straw compost, 5% (w/v) milled wheat straw). Cultures were grown on an orbital shaker (150 r.p.m.) at 30 °C for 8 weeks. The enrichment culture process will have favoured those species able to grow at 30 °C in a well aerated environment which are required characteristics for further experiments. As a result the isolated bacteria used in this study do not provide a full representation of the complex microbial community present in compost, but do represent a diverse collection of naturally coexisting isolates that could potentially be used in industrial bioprocessing. Each week serial dilutions were prepared and spread onto nutrient agar, potato dextrose agar and M9 minimal media containing 1.5% (w/v) agar and 1% (w/v) milled wheat straw. Single colonies that appeared morphologically distinct on agar plates (Supplementary Table 1) were assayed for activity against carboxymethylcellulose (CMC) and xylan (both from Sigma-Aldrich, Dorset, UK) using Congo red staining assays (Teather and Wood, 1982). Species with activity against CMC and/or xylan were identified by 16S rRNA gene sequencing (16S sequences were deposited in GenBank under the accession numbers KX527645-KX527656). The twelve species included in this study were chosen as they represent phylogenetic or functional diversity based on 16S rRNA sequences and CMC and xylan assays (Supplementary Figure 1; Supplementary Table 1).

### Biodiversity–ecosystem functioning experiment

Communities for the BEF experiment were designed using the random partition design described by Bell *et al.* (2009). Species were randomly divided into communities with species richness levels of 1, 2, 3, 4, 6 and 12 species with each isolate represented an equal number of times at each richness level. This process was repeated to produce 12 monocultures, 66 two-isolate communities, 60 three-isolate communities, 63 four-isolate communities, 66 six-isolate communities and one 12-isolate community. Each community was replicated five times to give a total of 1340 communities. The twelve species were grown for two days in 5 ml nutrient broth on an orbital shaker (150 r.p.m.) at 30 °C. Cultures were harvested by centrifugation, washed and suspended in M9 minimal media and left at room temperature for 2 h to metabolise remaining nutrients before OD_600_ was standardised to 0.1 to ensure similar starting densities. Deep well plates containing 380 μl M9 minimal media with 1% (w/v) milled wheat straw per well were inoculated with a total of 120 μl cultures, for example, monocultures were inoculated with 120 μl single species culture whereas the 12 species community was inoculated with 10 μl of each culture. The MicroResp system was used to measure respiration of cultures (Campbell *et al.*, 2003). Briefly, each well in the deep well plate is sealed to a microplate well containing indicator dye which changes colour in response to CO_2_ concentration. Microplates containing indicator gel were replaced every 24 h to prevent cultures becoming anaerobic. Community productivity was estimated as cumulative respiration (Tiunov and Scheu, 2005; Armitage, 2016). Specifically, cultures were grown for 7 days at 30 °C and productivity was measured as the cumulative change in absorbance (*λ*=595 nm) of the indicator gel immediately before and after being sealed to deep well cultures plates. The change in OD of the indicator gel from control wells containing no inoculum was used to account for atmospheric CO_2_ concentration. Note that due to the presence of particles of wheat straw in the growth medium it was not possible to measure change in microbial biomass by absorbance.

### Functional trait assays

To quantify the fundamental niche of each species, growth assays were performed on several polysaccharides present in lignocellulose. Hemicellulose substrates included xylan (Sigma-Aldrich), arabinoxylan (P-WAXYL, Megazyme, Bray, Ireland) and galactomannan (P-GALML, Megazyme); cellulose substrates included β-glucan (P-BGBL, Megazyme) and Whatman filter paper; additional substrates included pectin (Sigma-Aldrich) and Kraft lignin (Sigma-Aldrich). Cultures were prepared as described for the BEF experiment. These cultures (5 μl) were used to inoculate 495 μl of M9 minimal media with 0.2% (w/v) of each carbon source or one 6 mm sterile filter paper disc in 96-well deep well plates. Cultures were replicated six times and several blank wells containing no inoculum were included as negative controls. Cultures were grown for 7 days at 30 °C and the MicroResp system was used to measure culture respiration as described above.

### Statistical analysis

The biodiversity and ecosystem functioning relationship was analysed using the linear model method described by Bell *et al.* (2009). The species coefficients provided by this method give a measure of the effect of each species on community productivity relative to an average species: values of >1 indicate an above average contribution while values of <1 indicate a below average contribution to community productivity. To assess the effect of *Paenibacillus* sp. A8 and *Cellulomonas flavigena* D13 on community productivity, communities containing both species, *Paenibacillus* sp. A8 only, *C. flavigena* D13 only or neither of these species were compared using analysis of variance (ANOVA) followed by *post hoc* Tukey tests. Linear models were used to compare the ability of species richness and the presence or absence of *Paenibacillus* sp. A8 and *C. flavigena* D13 to predict community productivity.

To standardise measures of functional traits across diverse substrates, performance on each substrate was normalised by dividing by the maximum observed respiration on that substrate. For each bacterial isolate we can then calculate its fundamental niche (along the carbon degradation axis) by summing performance on all substrates. To estimate the niche space of each community we used the community niche (CN) metric described by Salles *et al.* (2009), which sums the maximal performance on each substrate: 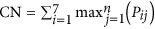, where *P*_*ij*_ is the performance of species *j* on carbon source *i* and *n* is the number of species in each community.

The ability of each functional trait to predict community productivity was analysed by summing performance of all species in a community on each carbon source to give a measure of the total fundamental niche space of that community. To approximate the realised niche space of communities we also assessed the ability of the maximum performance on each carbon source in a community to predict community productivity; this metric assumes that the species best able to grow on a given carbon source in a community dominates consumption of that carbon source providing a conservative estimate of realised niche. Linear regressions were used to analyse how well CN and functional trait performance predicted community productivity. It is important to note that because all species can grow on several carbon sources, summing functional trait use may act as a proxy of species richness. To control for this effect we analysed whether summed community functional traits remained significant when fitted to the residuals of the species richness model (that is, community productivity predicted by species richness). Competing models were compared using the Akaike information criterion.

## Results

### Biodiversity–ecosystem function relationship

**Figure 1:**
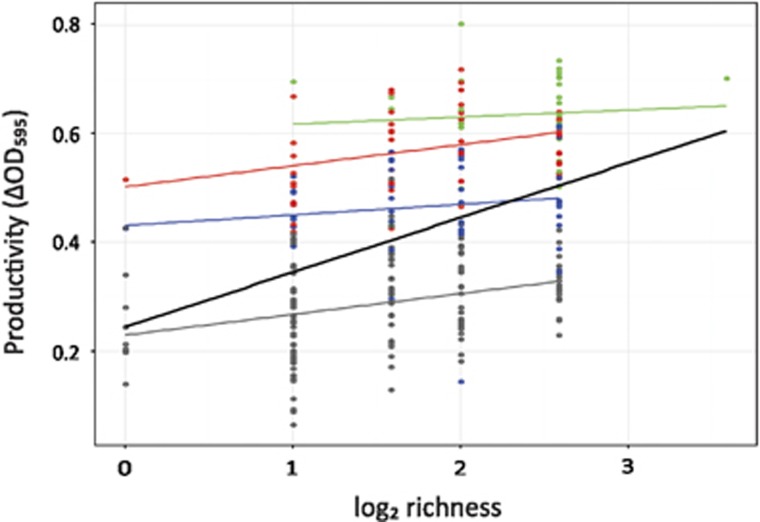
Relationship between community productivity and species richness. Black line shows linear regression for all data points (F_1, 264_=60.1, *R*^2^=0.19, *P*<0.001). Each point is the mean productivity of five replicate communities. Points are coloured by the presence or absence of *C. flavigena* D13 and *Paenibacillus* sp. A8 and linear regressions between community productivity and species richness are shown for each of these groups: green points represent communities containing both these species (F_1, 28_=0.42, *P*>0.05); red points represent communities containing *C. flavigena* D13 (F_1, 50_=4.43, *P*<0.05); blue points represent communities containing *Paenibacillus* sp. A8 (F_1, 50_=1.01, *P*>0.05); grey points represent communities containing neither of these species (F_1, 129_=60.1, *P*<0.001). Productivity is measured as the cumulative change in OD_595_ of MicroResp indicator plate after 7 days growth.

We observed a positive relationship between species richness and community productivity (F_1, 264_=60.1; *P*<0.001, Figure 1) with species richness explaining 19% of variation in productivity. As highlighted by the variance in productivity within species richness levels, species identity also had a significant effect on community productivity (F_12, 254_=45.3, *P*<0.001). The linear model coefficient for each species provides the estimated contribution of that species to community productivity relative to an average species (Bell *et al.*, 2009). Two species, *Paenibacillus* sp. A8 and *C. flavigena* D13, made significantly greater contributions to community function relative to an average species (F_1, 254_=73.1, *P*<0.001 and F_1, 254_=256.3, *P*<0.001 respectively, Supplementary Figure 2). Of the remaining species, the contribution of *Rheinheimera* sp. D14A and *Stenotrophomonas* sp. D12, did not significantly differ from the average species while the remaining eight species made significantly below average contributions to community functioning (Supplementary Figure 2).

To further investigate the effects of *Paenibacillus* sp. A8 and *C. flavigena* D13, the productivity of communities containing either one, both or neither of these species was compared. Communities that contained both *Paenibacillus* sp. A8 and *C. flavigena* D13 were significantly more productive than communities containing either one or neither of these species (*post hoc* Tukey tests, *P*<0.001, Figure 1). The productivity of communities containing both *Paenibacillus* sp. A8 and *C. flavigena* D13 did not significantly differ across species richness levels suggesting additional species within these communities are not contributing to community productivity (F_1, 28_=0.42, *P*>0.05, green line Figure 1). Communities containing only *C. flavigena* D13 were more productive than those containing only *Paenibacillus* sp. A8 (*post hoc* Tukey test, *P*<0.001), while communities which did not contain these species were significantly less productive than communities containing either one of these species (*post hoc* Tukey test, *P*<0.001). These results indicate that the positive BEF relationship is predominantly driven by the selection effect, that is, more diverse communities are more likely to contain the highly performing species *Paenibacillus* sp. A8 and *C. flavigena* D13 and are therefore more productive.

### Quantification of functional traits

**Figure 2:**
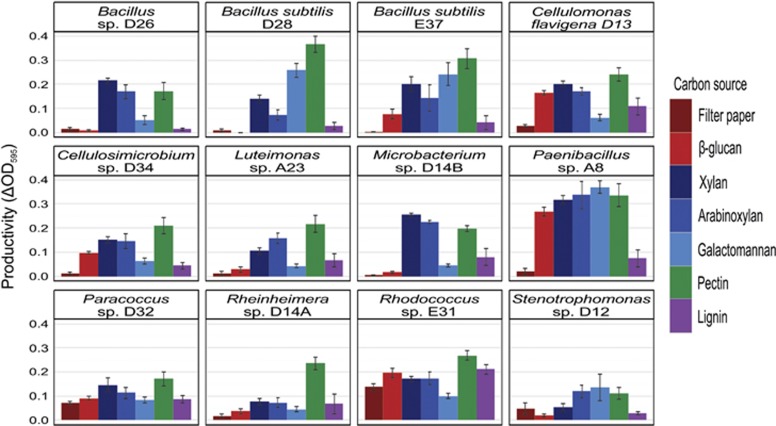
Productivity of species grown on each carbon source. Filter paper and β-glucan represent cellulose like substrates (red); xylan, arabinoxylan and galactomannan represent hemicelluloses (blue). Productivity is measured as the cumulative change in OD of MicroResp indicator plates over 7 days.

To determine if differences in productivity could be explained by the functional traits of species we assayed the ability of species to utilise various components of lignocellulose. All species were able to grow to varying degrees on the labile substrates, hemicellulose (xylan, arabinoxylan and galactomannan) and pectin, whereas growth on recalcitrant substrates (β-glucan, filter paper and lignin) was less universal (Figure 2). This pattern is consistent with the hypothesis that functional groups that degrade recalcitrant substrates are not as common as those that degrade labile substrates (Schimel and Gulledge, 1998; Waldrop and Firestone, 2004). A linear model revealed significant main effects of both species (F_11,336_=30.3, *P*<0.001) and carbon source (F_6,336_=105.8, *P*<0.001) on productivity and a significant interaction between these factors (F_66, 336_=6.8, *P*<0.001), suggesting niche differentiation in resource use among the species. It is notable that some species, in particular *Rhodococcus* sp. E31, displayed generalist resource use, being able to grow on recalcitrant substrates like lignin as well as on the more labile substrates.

### Community productivity and functional traits

**Figure 3:**
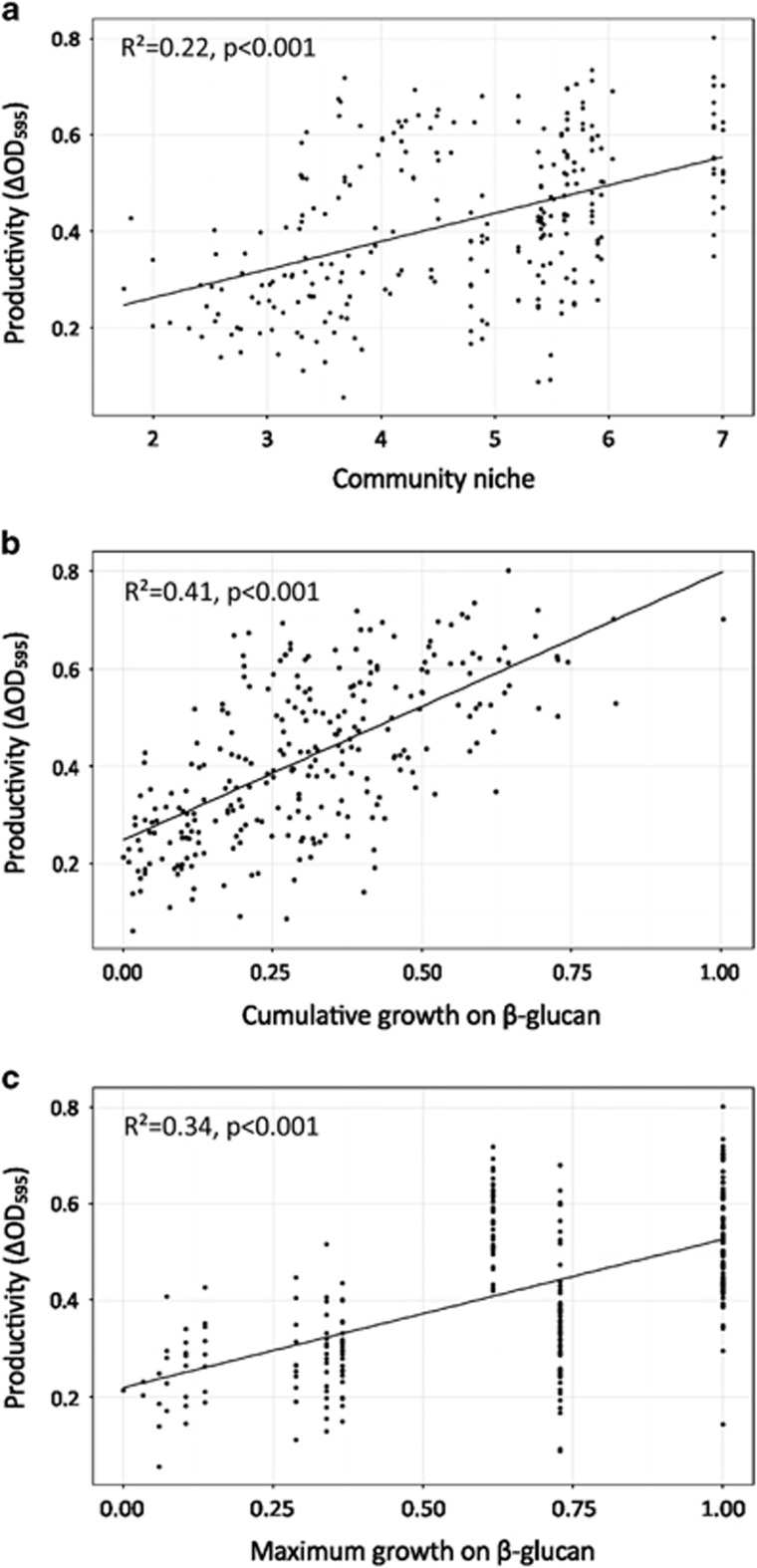
Relationship between community productivity and (**a**) community niche, (**b**) cumulative ability of constituent species to utilise β-glucan and (**c**) maximum ability of constituent species to utilise β-glucan. Higher community niche indicates communities can utilise more resources more efficiently. The ability of constituent species to utilise β-glucan was calculated from their ability to grow on this substrate in functional trait assays (Figure 2). Each point represents the mean productivity of five replicate communities.

To determine if the functional niche of communities could be used to predict productivity we calculated community niche as described by Salles *et al.* (2009). This index sums the maximum growth achieved by a constituent species on each substrate. We found a significant positive relationship between community niche and community productivity (F_1, 264_=73.31, *P*<0.001, Figure 3a). Similar to the results of Salles *et al.* (2009), community niche explained more variation in community productivity than species richness (22% and 19%, respectively).

When calculating community niche, each functional trait is weighted equally despite differences in the abundances of substrates in wheat straw lignocellulose, for example, cellulose constitutes 40–50% whereas pectin only constitutes 1–2%. To determine which functional traits were important for predicting community productivity we summed the growth of constituent species on each carbon source used in functional trait assays to calculate the total fundamental niche of that community. The summed activity on β-glucan had a significant positive relationship with productivity (F_1, 264_=182.7, *P*<0.001) and was the best predictor of community productivity, explaining 41% of variation (Figure 3b). The ability to utilise arabinoxylan and xylan also had significant positive relationships with productivity (F_1, 264_=105.8, *P*<0.001 and F_1, 264_=98.6, *P*<0.001, respectively), explaining 29% and 27% of variation, respectively. There were significant positive relationships between the remaining carbon sources and community productivity though these explained less variation than community richness and were not significant when species richness was included in models. The fundamental niche space of community is unlikely to be achieved due to interactions between species such as competition for resources. Therefore to approximate the realised niche space of each community we also analysed the maximum performance per carbon source in a community. Consistent with the analysis of summed performance, maximum performance on β-glucan, arabinoxylan and xylan had significant positive relationships with productivity (F_1, 264_=134.8, *P*<0.001, F_1, 264_=76.2, *P*<0.001 and F_1, 264_=74.5, *P*<0.001, respectively) explaining 34%, 23% and 22% of variation, respectively. There were significant positive relationships between the maximum performance on lignin (F_1, 264_=7.4, *P*<0.01), pectin (F_1, 264_=20.8, *P*<0.001) and galactomannan (F_1, 264_=47.1, *P*<0.001) and community productivity though these explained less variation than community richness. There was no significant relationship between the maximum ability to degrade filter paper and community productivity (Supplementary Table 2). This suggests that identifying and measuring key functional traits could be a better predictor of community productivity than either species richness or community niche.

## Discussion

Understanding the factors that influence microbial community productivity has potentially important ecological and industrial applications (Widder *et al.*, 2016). The ability of community niche to predict functioning in well-defined media has been demonstrated previously (Salles *et al.*, 2009). Here, we define for communities growing in complex undefined media, the key functional resource use traits that predict decomposer community productivity. Crucially, functional resource use traits explained more variation in productivity than either species richness or measures of community niche. Indeed, a single function, the ability to degrade β-glucan, explained a larger proportion of variation than community niche. This key functional trait was shared by two dominant strains which were shown to significantly increase the productivity of communities.

As with several previous BEF studies (Bell *et al.*, 2009; Gravel *et al.*, 2011; Awasthi *et al.*, 2014), we identified a positive relationship between species richness and community productivity. By analysing the effect of community composition we found that the presence of two highly functioning species, *Paenibacillus* sp. A8 and *C. flavigena* D13, significantly increased community productivity suggesting this positive BEF relationship is driven by the selection effect. To determine if the dominance of these two species could be explained by their functional traits, we compared the ability of these species to utilise the various carbon sources used in functional trait assays to the other species. With the exception of *Rhodococcus* sp. E31, *Paenibacillus* sp. A8 and *C. flavigena* D13 were the highest performing species on β-glucan (Figure 2). The ability to utilise β-glucan may suggest these species are able to metabolise the cellulose portion of wheat straw in addition to the more labile hemicellulose and pectin fractions. Interestingly, when the productivity of communities containing either one, both or neither of these species is compared across each day of the experiment (Supplementary Figure 3), it is noticeable that communities containing neither of these species have very low productivity during the later days of the experiment. A possible explanation is that easily accessible labile substrates are being used within the first two days of growth after which only recalcitrant and inaccessible substrates remain. The ability to degrade cellulose would allow *Paenibacillus* sp. A8 and *C. flavigena* D13 to maintain higher levels of growth when labile substrates become depleted.

Interestingly, *Paenibacillus* sp. A8 and *C. flavigena* D13 have similar functional traits which would indicate they occupy overlapping niche space and may be in direct competition with each other. However, communities containing both these species were significantly more productive than communities containing only one or neither suggesting complementarity or facilitation effect between these species, that is, they are able to exploit a wider niche space when grown together potentially because they each produce enzymes or by-products that improve the overall community productivity. Wohl *et al.* (2004) found a similar result whereby functionally redundant cellulose degrading bacteria were more productive in communities than in monoculture.

The ability of species within communities to utilise β-glucan was a better predictor of community productivity than measures of community niche or species richness. The significance of this activity is consistent with the composition of wheat straw lignocellulose, which is made up of 40–50% cellulose. Interestingly, functional trait assays revealed that *Rhodococcus* sp. E31 achieved the second highest growth on β-glucan but this species did not significantly increase community productivity compared to an average species (Supplementary Figure 2). In addition, *Rhodococcus* sp. E31 was able to utilise lignin as well as the more labile hemicellulose substrates (Figure 2). It might have reasonably been expected that as lignin is the major contributing factor to recalcitrance, species able to degrade it would increase community productivity by increasing accessibility of saccharification enzymes to cellulose. The limited contribution of *Rhodococcus* sp. E31 to community productivity may be explained in part by structural differences between Kraft lignin used in functional trait assays and native lignin present in lignocellulose (Vishtal and Kraslawski, 2011). Alternatively, although able to achieve efficient degradation of all substrates in monoculture growth assays, *Rhodococcus* sp. E31 may be outcompeted in communities and unable to achieve the functional potentials revealed by trait assays. Recalcitrant substrates may require more energy expensive breakdown pathways than labile substrates (Lynd *et al.*, 2002) which may put species that are specialised to degrade such substrates, for example, *Rhodococcus* sp. E31, at a competitive disadvantage in communities. Measuring the abundance of species in each community would allow us to better determine the functional traits present in communities assuming that enzyme expression does not differ between monoculture and communities. Alternatively, it may be possible to match functional traits to community productivity by comparing the transcriptome and proteome of focal communities, although any such approach is necessarily limited by the correct annotation of functional genes and/or proteins.

Rivett *et al.* (2016) found that the ability of species to degrade labile resources could be explained by metabolic plasticity whereas the ability to degrade more recalcitrant substrates required evolutionary adaptation. Species best adapted to utilise the accessible labile substrates may be able to dominate communities during initial growth stages but as labile substrates become depleted, species able to adapt to utilise the remaining recalcitrant substrates will become more dominant in communities. When comparing the contribution of species across each day of the BEF experiment, we found that the contribution of species did not noticeably differ throughout the seven days of growth. *Paenibacillus* sp. A8 significantly improved community productivity relative to the average species on each day while *C. flavigena* D13 made a significantly higher contribution than the average species from day 2 onwards (Supplementary Figure 3). The presence of *Rheinheimera* sp. D14A made a significantly above average contribution to community productivity on day one of the experiment, though for the remaining 6 days the contribution of this species did not significantly differ from that of an average species. Of the remaining 9 species, contributions remained lower than or did not significantly differ from the average species throughout the 7 days. The ability of *C. flavigena* D13 and *Paenibacillus* sp. A8 to efficiently degrade both recalcitrant and labile substrates may allow them to outcompete other species before they are able to adapt to utilise recalcitrant substrates. Allowing the species used here a period of evolutionary adaptation to the wheat straw substrate may increase their ability to degrade recalcitrant substrates and alter the dominance hierarchy within these communities and is an interesting topic for future study.

In conclusion, we have identified key functional traits that define the productivity of communities degrading lignocellulose. We found that the degradative abilities of communities against β-glucan, arabinoxylan and xylan were able to predict community productivity more effectively than either measures of community niche or species richness. Furthermore, we found that two species, *Paenibacillus* sp. A8 and *C. flavigena* D13, made greater than average contributions to community productivity suggesting a key role for the selection effect in driving the observed positive BEF relationship. Our results suggest that, using simple experiments, it is possible to identify the important functional traits and species that drive microbial community productivity on complex natural substrates like wheat straw, potentially simplifying efforts to predict the functioning of natural communities and the assembly of highly performing communities for biotechnological industrial applications.

## References

[bib1] Armitage DW. (2016). Time-variant species pools shape competitive dynamics and biodiversity–ecosystem function relationships. Proc R Soc B 283: 20161437. 10.1098/rspb.2016.1437 PMC5031662 27629035

[bib2] Awasthi A, Singh M, Soni SK, Singh R, Kalra A. (2014). Biodiversity acts as insurance of productivity of bacterial communities under abiotic perturbations. ISME J 8: 2445–2452. 24926862 10.1038/ismej.2014.91 PMC4260711

[bib3] Bell T, Lilley AK, Hector A, Schmid B, King L, Newman JA et al. (2009). A linear model method for biodiversity–ecosystem functioning experiments. Am Nat 174: 836–849. 19842969 10.1086/647931

[bib4] Bell T, Newman JA, Silverman BW, Turner SL, Lilley AK. (2005). The contribution of species richness and composition to bacterial services. Nature 436: 1157–1160. 16121181 10.1038/nature03891

[bib5] Bonkowski M, Roy J. (2005). Soil microbial diversity and soil functioning affect competition among grasses in experimental microcosms. Oecologia 143: 232–240. 15703913 10.1007/s00442-004-1790-1

[bib6] Brune A. (2014). Symbiotic digestion of lignocellulose in termite guts. Nat Rev Microbiol 12: 168–180. 24487819 10.1038/nrmicro3182

[bib7] Campbell CD, Chapman SJ, Cameron CM, Davidson MS, Potts JM. (2003). A rapid microtiter plate method to measure carbon dioxide evolved from carbon substrate amendments so as to determine the physiological profiles of soil microbial communities by using whole soil. Appl Environ Microbiol 69: 3593–3599. 12788767 10.1128/AEM.69.6.3593-3599.2003 PMC161481

[bib8] Cydzik-Kwiatkowska A, Zielińska M. (2016). Bacterial communities in full-scale wastewater treatment systems. World J Microbiol Biotechnol 32: 66. 26931606 10.1007/s11274-016-2012-9 PMC4773473

[bib9] Elsas JD van, Chiurazzi M, Mallon CA, Elhottovā D, Krištůfek V, Salles JF. (2012). Microbial diversity determines the invasion of soil by a bacterial pathogen. Proc Natl Acad Sci USA 109: 1159–1164. 22232669 10.1073/pnas.1109326109 PMC3268289

[bib10] Gravel D, Bell T, Barbera C, Bouvier T, Pommier T, Venail P et al. (2011). Experimental niche evolution alters the strength of the diversity-productivity relationship. Nature 469: 89–U1601. 21131946 10.1038/nature09592

[bib11] Himmel ME, Ding S-Y, Johnson DK, Adney WS, Nimlos MR, Brady JW et al. (2007). Biomass recalcitrance: engineering plants and enzymes for biofuels production. Science 315: 804–807. 17289988 10.1126/science.1137016

[bib12] Hooper DU, Chapin FS, Ewel JJ, Hector A, Inchausti P, Lavorel S et al. (2005). Effects of biodiversity on ecosystem functioning: a consensus of current knowledge. Ecol Monogr 75: 3–35.

[bib13] Johnson DR, Helbling DE, Lee TK, Park J, Fenner K, Kohler H-PE et al. (2015). Association of biodiversity with the rates of micropollutant biotransformations among full-scale wastewater treatment plant communities. Appl Environ Microbiol 81: 666–675. 25398862 10.1128/AEM.03286-14 PMC4277575

[bib14] Jorgensen H, Kristensen JB, Felby C. (2007). Enzymatic conversion of lignocellulose into fermentable sugars: challenges and opportunities. Biofuels Bioprod Biorefining-Biofpr 1: 119–134.

[bib15] Krause S, Le Roux X, Niklaus PA, Van Bodegom PM, Lennon JT, Bertilsson S et al. (2014). Trait-based approaches for understanding microbial biodiversity and ecosystem functioning. Aquat Microbiol 5: 251. 10.3389/fmicb.2014.00251 PMC4033906 24904563

[bib16] Langenheder S, Bulling MT, Prosser JI, Solan M. (2012). Role of functionally dominant species in varying environmental regimes: evidence for the performance-enhancing effect of biodiversity. BMC Ecol 12: 14. 22846071 10.1186/1472-6785-12-14 PMC3480835

[bib17] Langenheder S, Bulling MT, Solan M, Prosser JI. (2010). Bacterial biodiversity-ecosystem functioning relations are modified by environmental complexity. PLoS One 5: e10834. 20520808 10.1371/journal.pone.0010834 PMC2877076

[bib18] Liao JC, Mi L, Pontrelli S, Luo S. (2016). Fuelling the future: microbial engineering for the production of sustainable biofuels. Nat Rev Microbiol 14: 288–304. 27026253 10.1038/nrmicro.2016.32

[bib19] Lopez-Gonzalez JA, Vargas-Garcia M, del C, Lopez MJ, Suarez-Estrella F, Jurado M et al. (2014). Enzymatic characterization of microbial isolates from lignocellulose waste composting: chronological evolution. J Environ Manage 145: 137–146. 25026369 10.1016/j.jenvman.2014.06.019

[bib20] Lynd LR, Weimer PJ, Zyl WH van, Pretorius IS. (2002). Microbial cellulose utilization: fundamentals and biotechnology. Microbiol Mol Biol Rev 66: 739–739. 10.1128/MMBR.66.3.506-577.2002 PMC120791 12209002

[bib21] McGuire KL, Treseder KK. (2010). Microbial communities and their relevance for ecosystem models: decomposition as a case study. Soil Biol Biochem 42: 529–535.

[bib22] Mokany K, Ash J, Roxburgh S. (2008). Functional identity is more important than diversity in influencing ecosystem processes in a temperate native grassland. J Ecol 96: 884–893.

[bib23] Naik SN, Goud VV, Rout PK, Dalai AK. (2010). Production of first and second generation biofuels: a comprehensive review. Renew Sustain Energy Rev 14: 578–597.

[bib24] Pauly M, Keegstra K. (2008). Cell-wall carbohydrates and their modification as a resource for biofuels. Plant J 54: 559–568. 18476863 10.1111/j.1365-313X.2008.03463.x

[bib25] Rivett DW, Scheuerl T, Culbert CT, Mombrikotb SB, Johnstone E, Barraclough TG et al. (2016). Resource-dependent attenuation of species interactions during bacterial succession. ISME J 10: 2259–2268. 26894447 10.1038/ismej.2016.11 PMC4989303

[bib26] Salles JF, Poly F, Schmid B, Roux XL. (2009). Community niche predicts the functioning of denitrifying bacterial assemblages. Ecology 90: 3324–3332. 20120802 10.1890/09-0188.1

[bib27] Schimel JP, Gulledge J. (1998). Microbial community structure and global trace gases. Glob Change Biol 4: 745–758.

[bib28] Singh M, Awasthi A, Soni SK, Singh R, Verma RK, Kalra A. (2015). Complementarity among plant growth promoting traits in rhizospheric bacterial communities promotes plant growth. Sci Rep 5: 15500. 26503744 10.1038/srep15500 PMC4621411

[bib29] Soliveres S, van der Plas F, Manning P, Prati D, Gossner MM, Renner SC et al. (2016). Biodiversity at multiple trophic levels is needed for ecosystem multifunctionality. Nature 536: 456–459. 27533038 10.1038/nature19092

[bib30] Teather RM, Wood PJ. (1982). Use of Congo red-polysaccharide interactions in enumeration and characterization of cellulolytic bacteria from the bovine rumen. Appl Environ Microbiol 43: 777–780. 7081984 10.1128/aem.43.4.777-780.1982 PMC241917

[bib31] Tiunov AV, Scheu S. (2005). Facilitative interactions rather than resource partitioning drive diversity-functioning relationships in laboratory fungal communities. Ecol Lett 8: 618–625.

[bib32] Van Der Heijden MGA, Bardgett RD, Van Straalen NM. (2008). The unseen majority: soil microbes as drivers of plant diversity and productivity in terrestrial ecosystems. Ecol Lett 11: 296–310. 18047587 10.1111/j.1461-0248.2007.01139.x

[bib33] Vishtal A, Kraslawski A. (2011). Challenges in industrial applications of technical lignins. Bioresources 6: 3547–3568.

[bib34] Waldrop MP, Firestone MK. (2004). Microbial community utilization of recalcitrant and simple carbon compounds: impact of oak-woodland plant communities. Oecologia 138: 275–284. 14614618 10.1007/s00442-003-1419-9

[bib35] Wei H, Tucker MP, Baker JO, Harris M, Luo Y, Xu Q et al. (2012). Tracking dynamics of plant biomass composting by changes in substrate structure, microbial community, and enzyme activity. Biotechnol Biofuels 5: 20. 22490508 10.1186/1754-6834-5-20 PMC3384452

[bib36] Widder S, Allen RJ, Pfeiffer T, Curtis TP, Wiuf C, Sloan WT et al. (2016). Challenges in microbial ecology: building predictive understanding of community function and dynamics. ISME J 10: 2557–2568. 27022995 10.1038/ismej.2016.45 PMC5113837

[bib37] Wohl DL, Arora S, Gladstone JR. (2004). Functional redundancy supports biodiversity and ecosystem function in a closed and constant environment. Ecology 85: 1534–1540.

