# Supplementary material for: Defining the functional traits that drive bacterial decomposer community productivity
Source: ISME J. 2017 Mar 21;11(7):1680–7. doi: 10.1038/ismej.2017.22 (PMC5480597; doi:10.1038/ismej.2017.22)
Supplement: Supplementary Information [file ismej201722x1.doc]

**Supplementary information**

**Supplementary Table 1 –** Colony morphology and activity of isolates on xylan and carboxymethylcellulose (CMC) plate assays. Clear halos indicate enzymatic activity against xylan or CMC.

| **Isolate** | **Colony morphology** | **Xylan activity assay** | **CMC activity assay** |
| --- | --- | --- | --- |
| *Cellulomonas flavigena* sp. D13 | 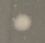 | 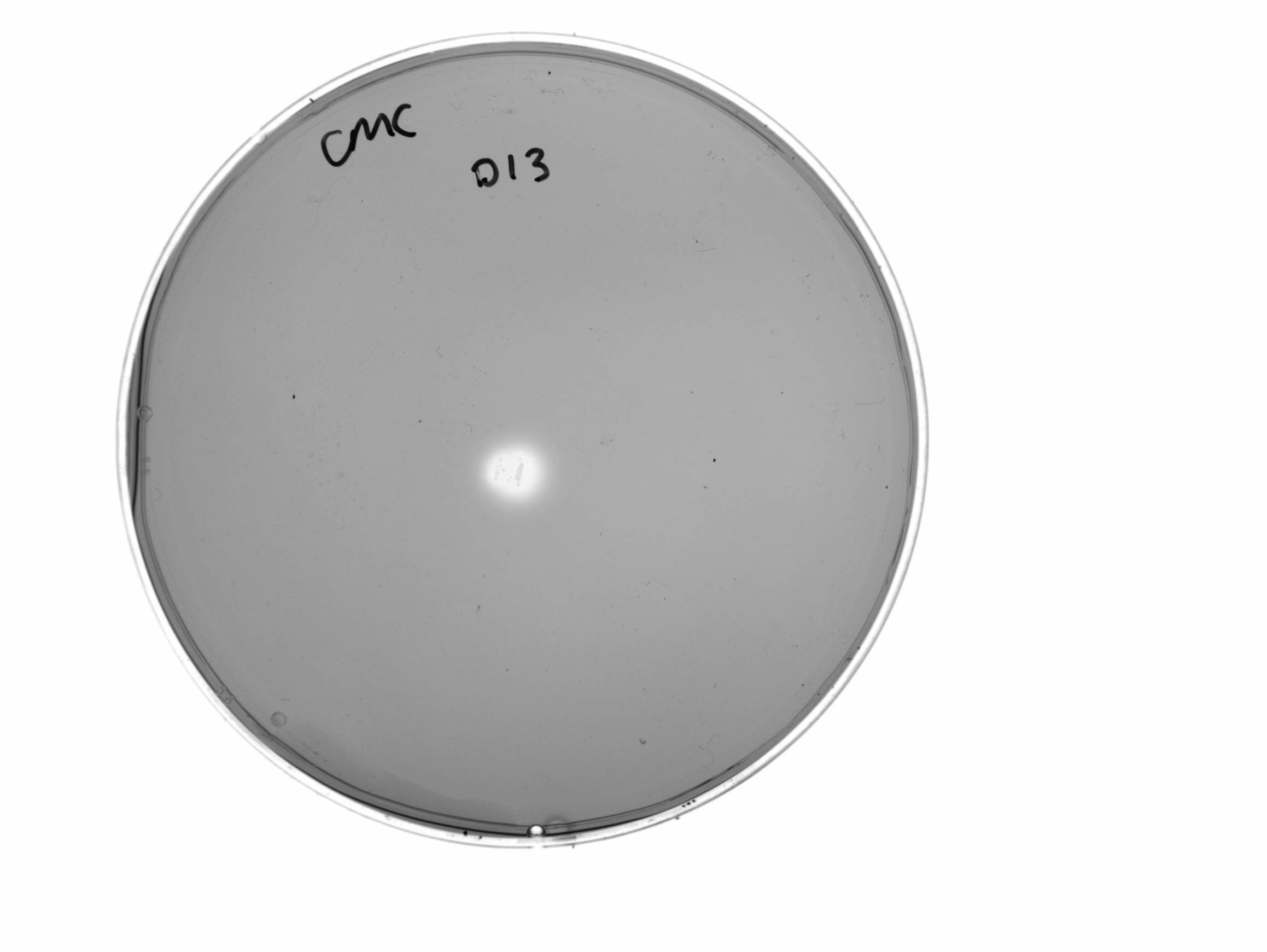 | 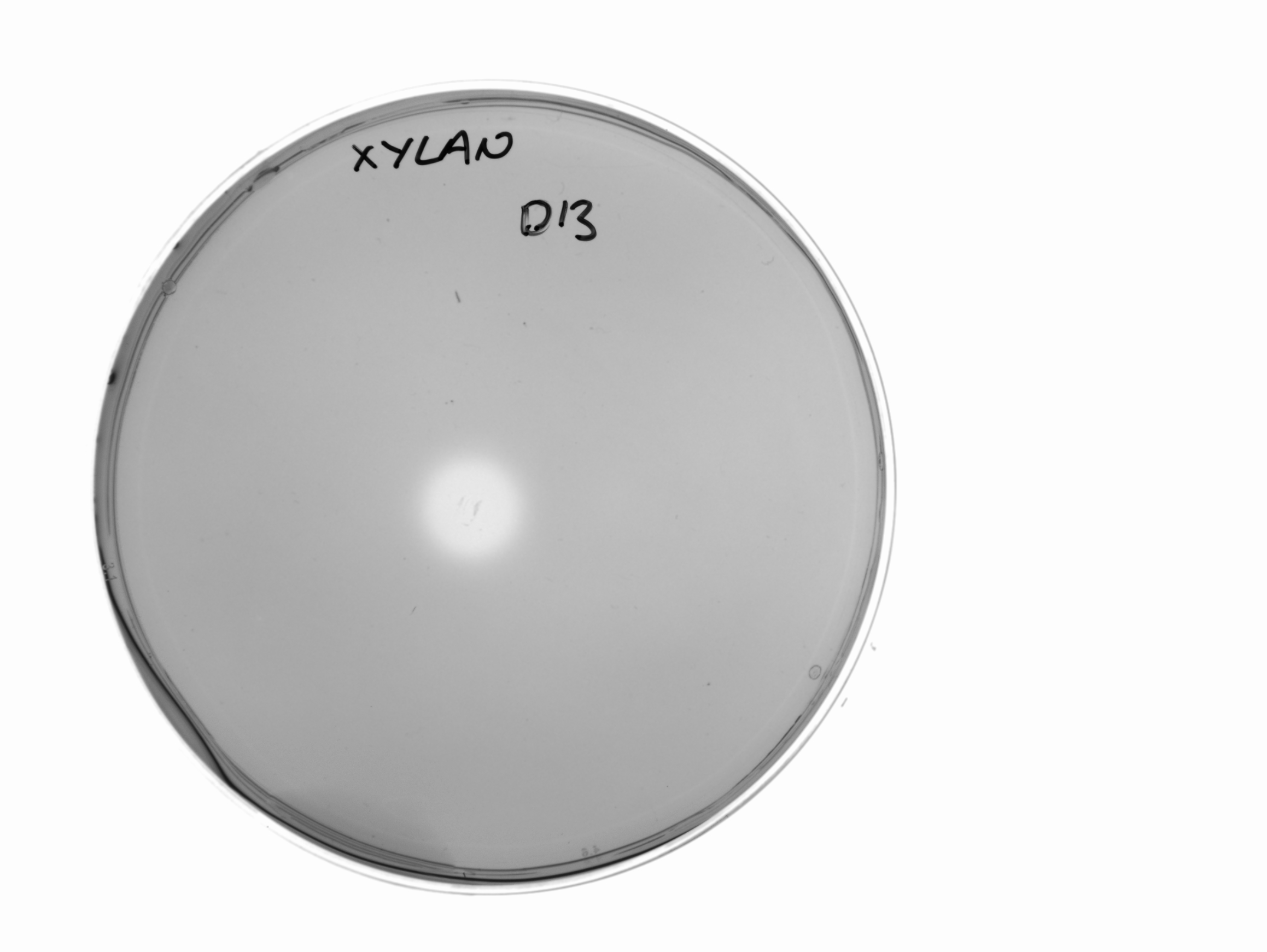 |
| *Cellulosimicrobium* sp. D34 | 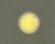 | 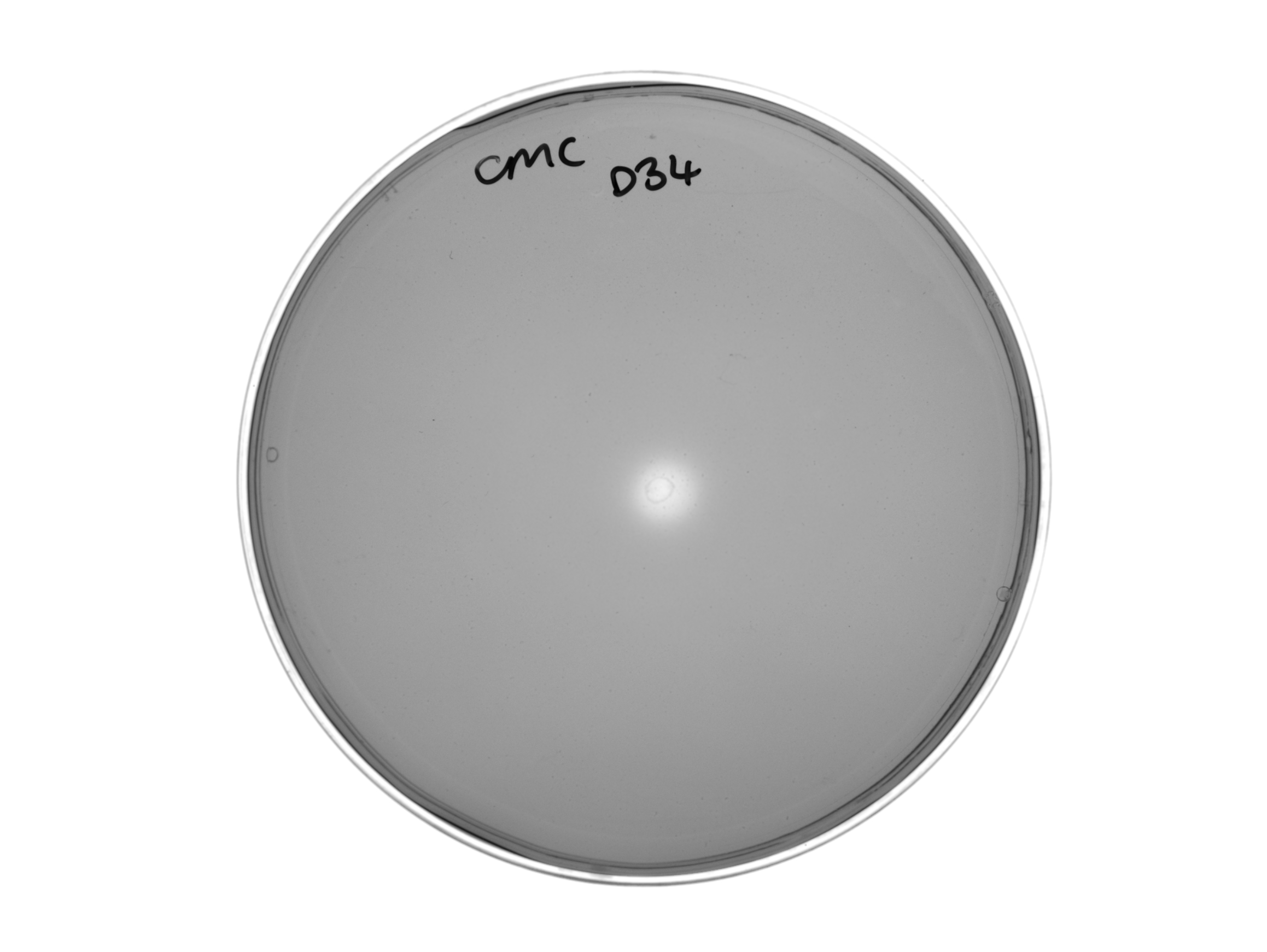 | 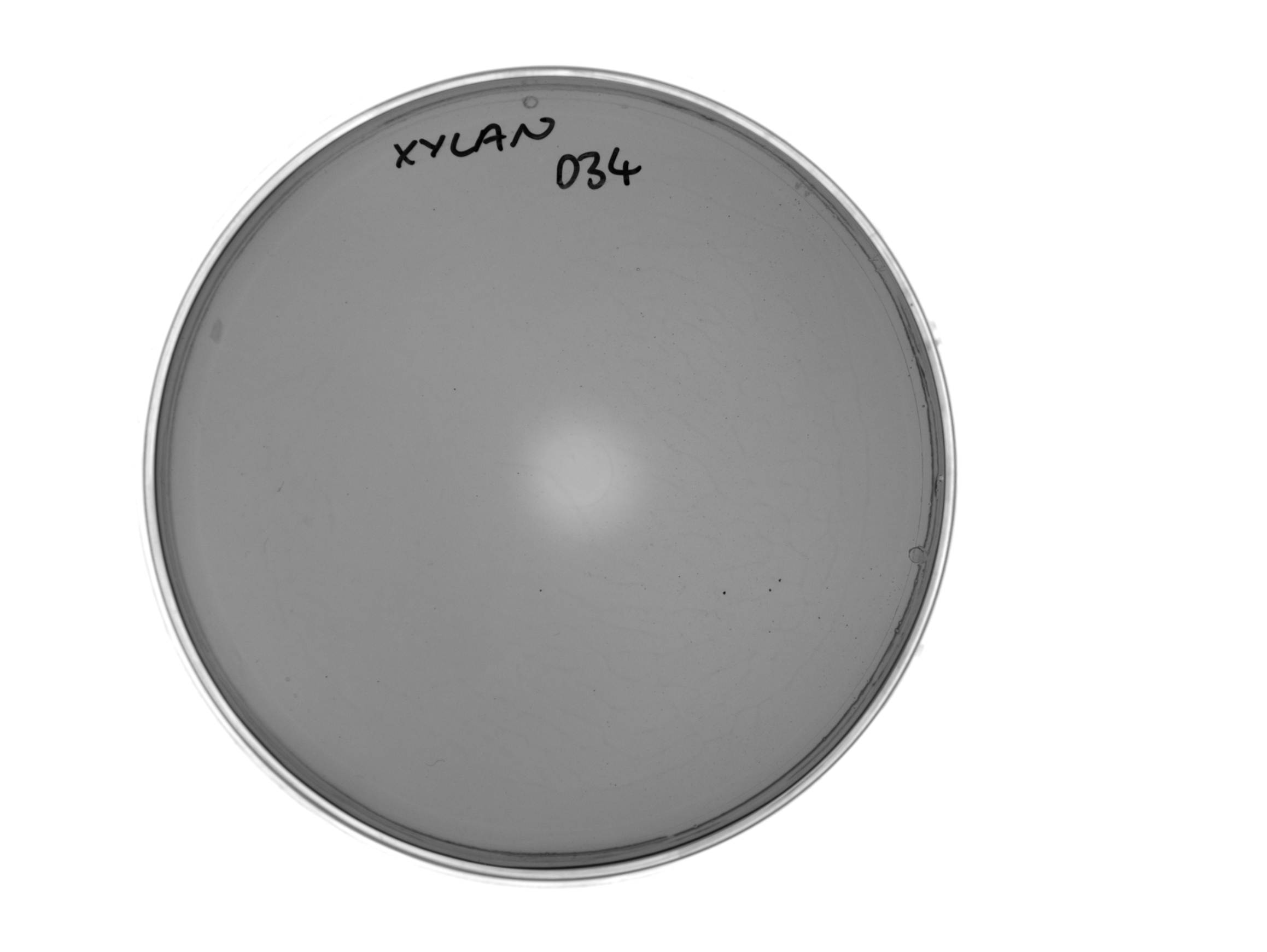 |
| *Microbacterium* sp. D14B | 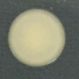 | 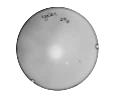 | 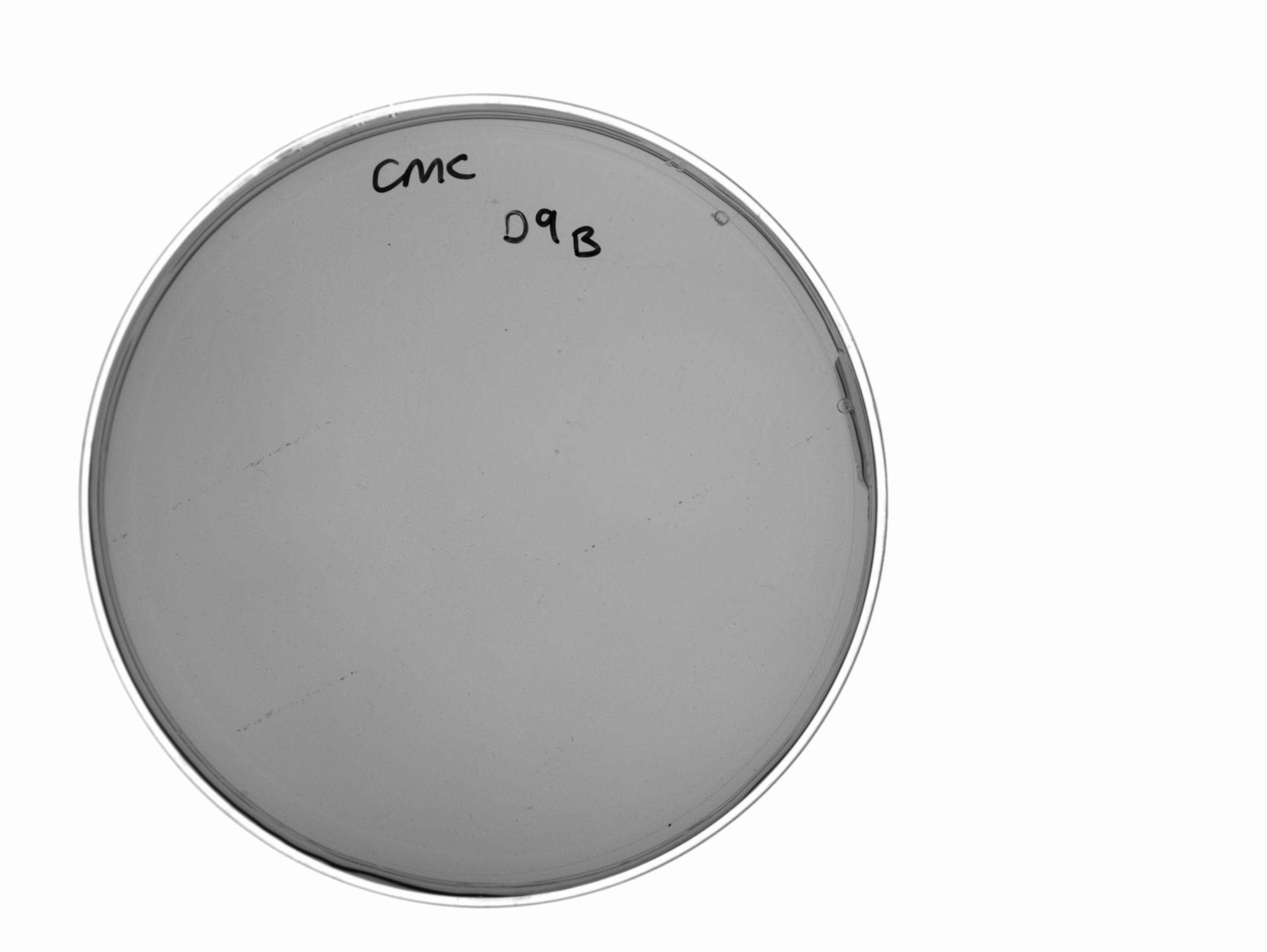 |
| *Rhodococcus* sp. E31 | 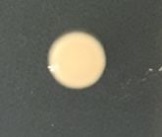 | 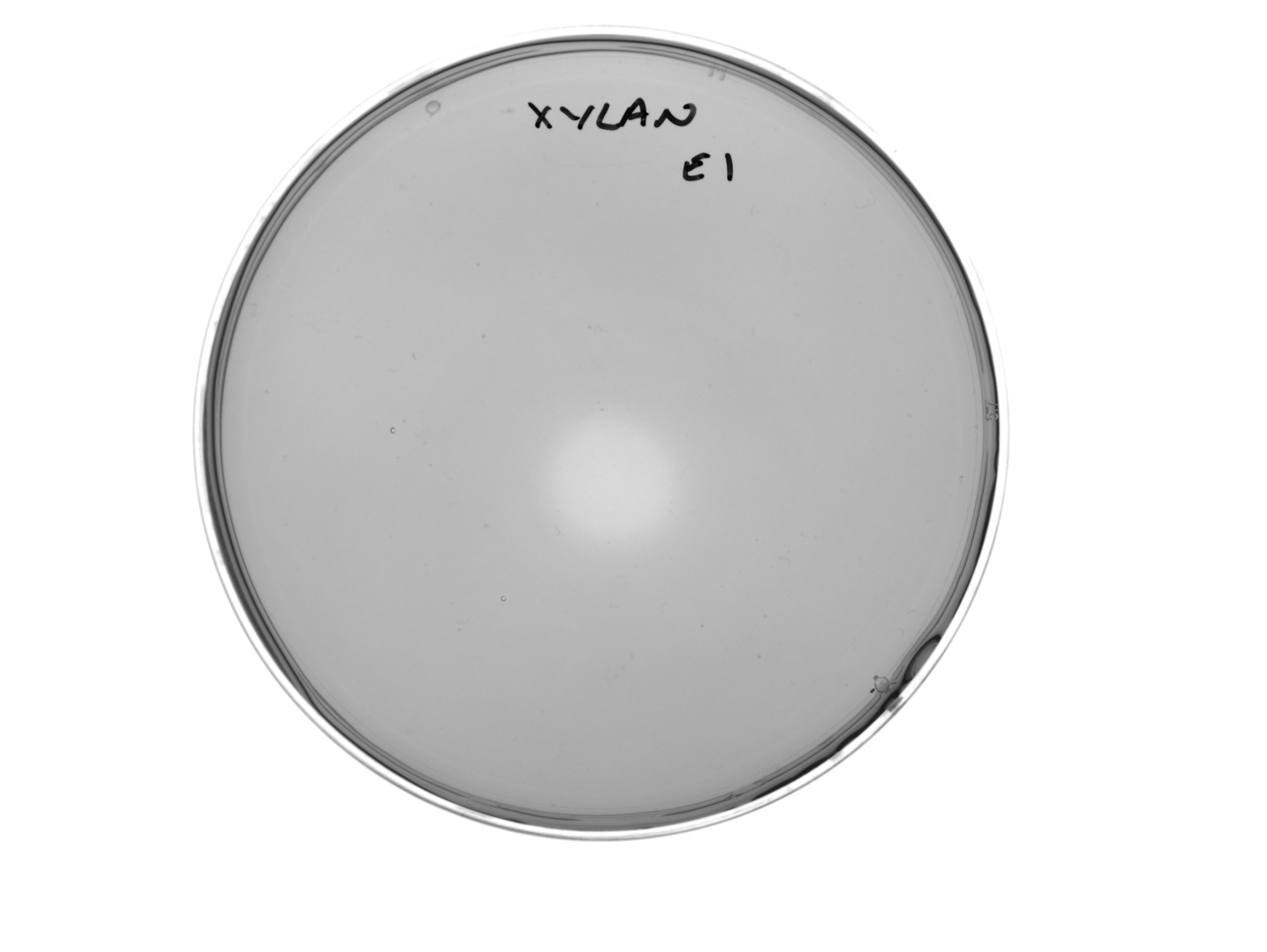 | 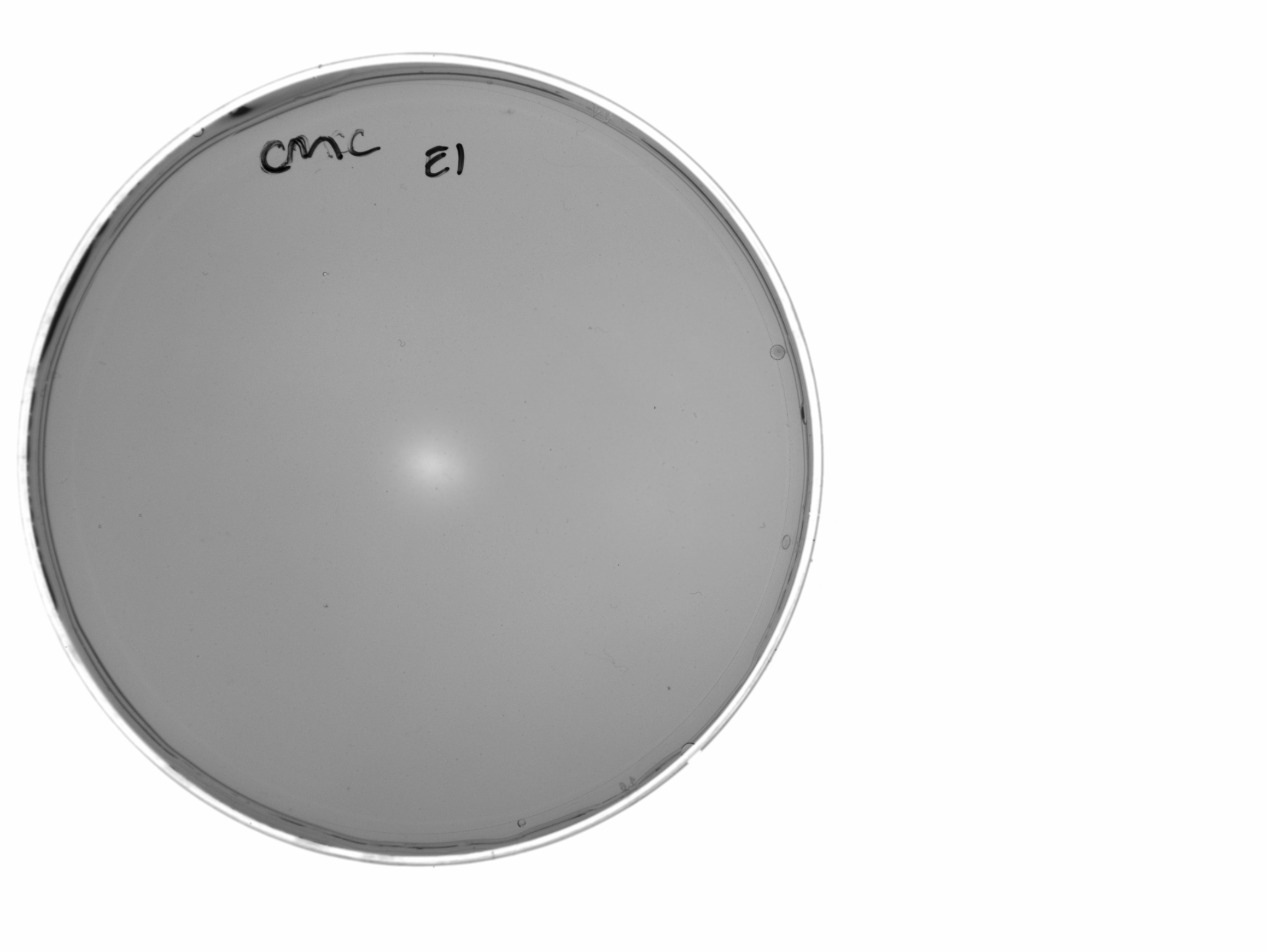 |
| *Paenibacillus* sp. A8 | 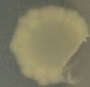 | 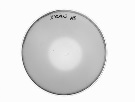 | 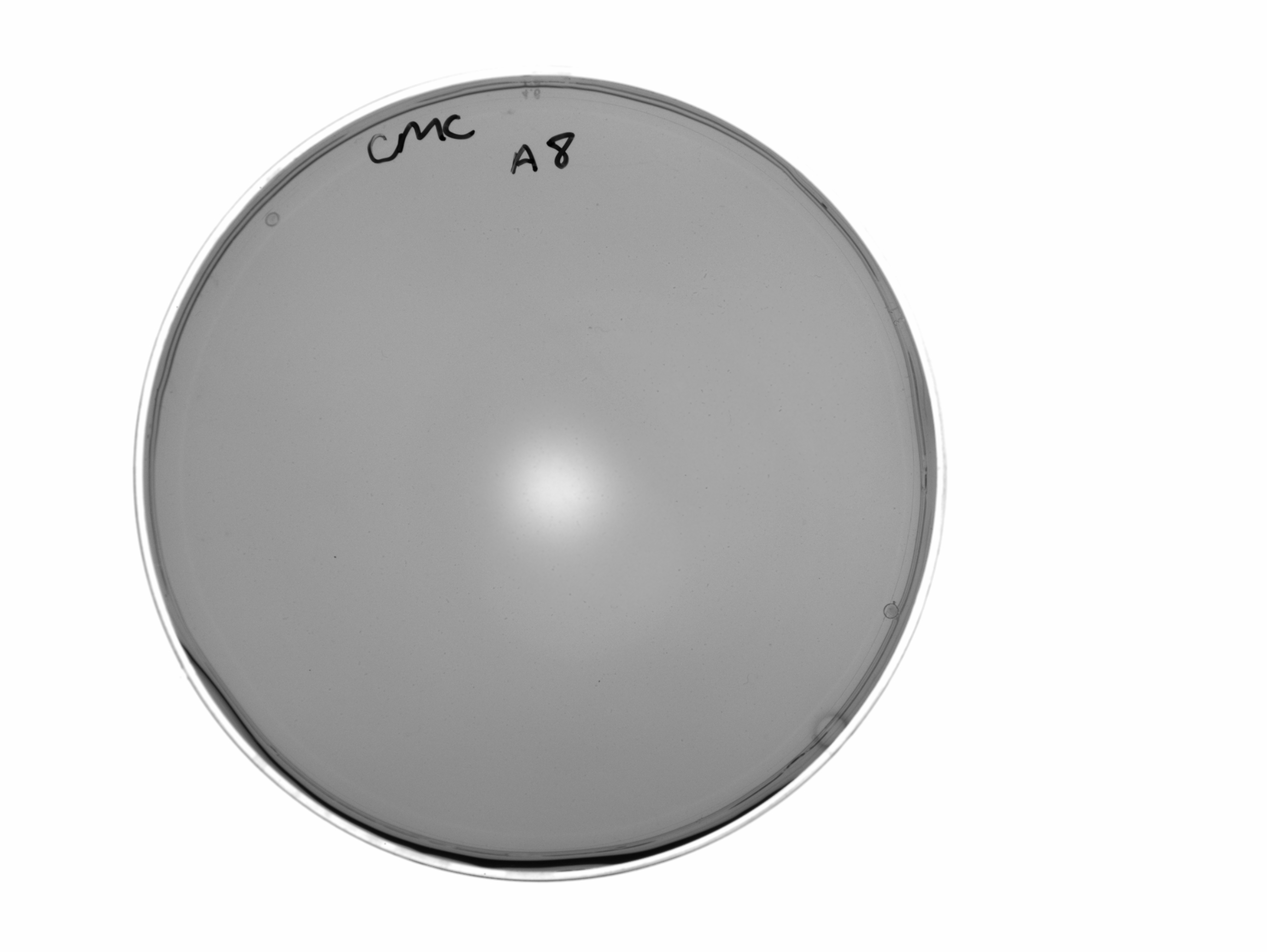 |
| *Bacillus* sp. D26 | 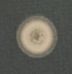 | 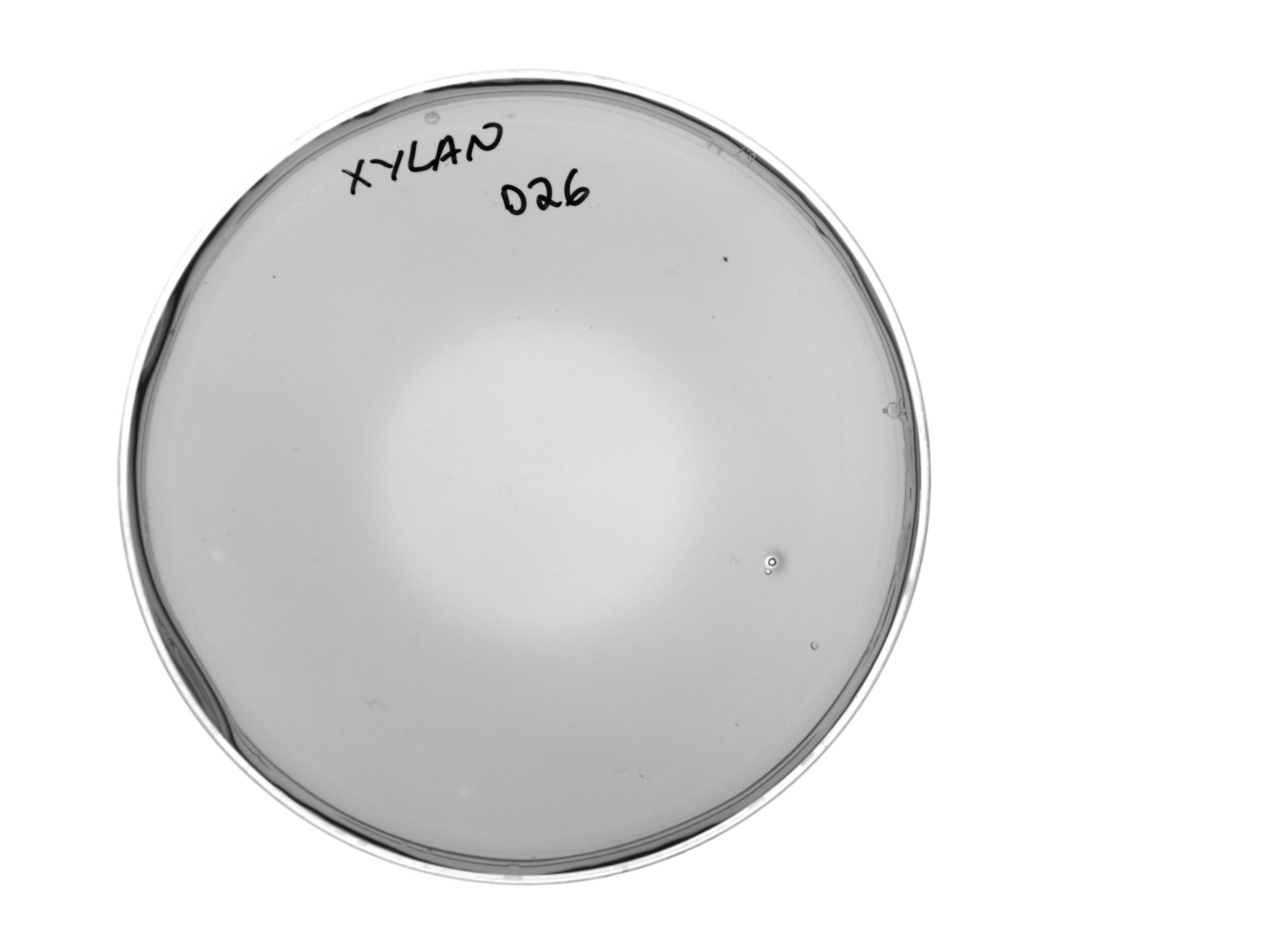 | 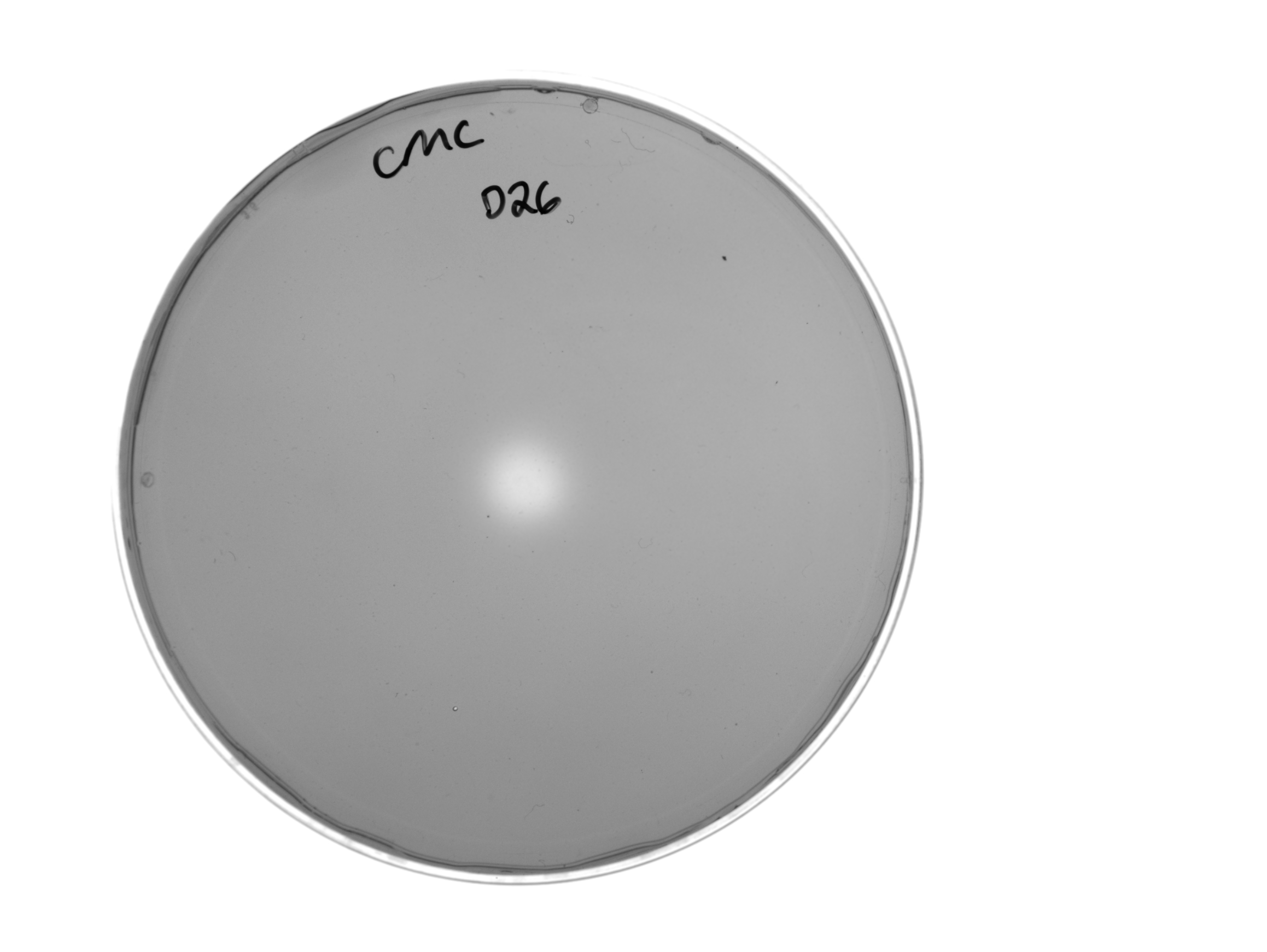 |
| *Bacillus* sp. D28 | 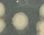 | 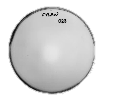 | 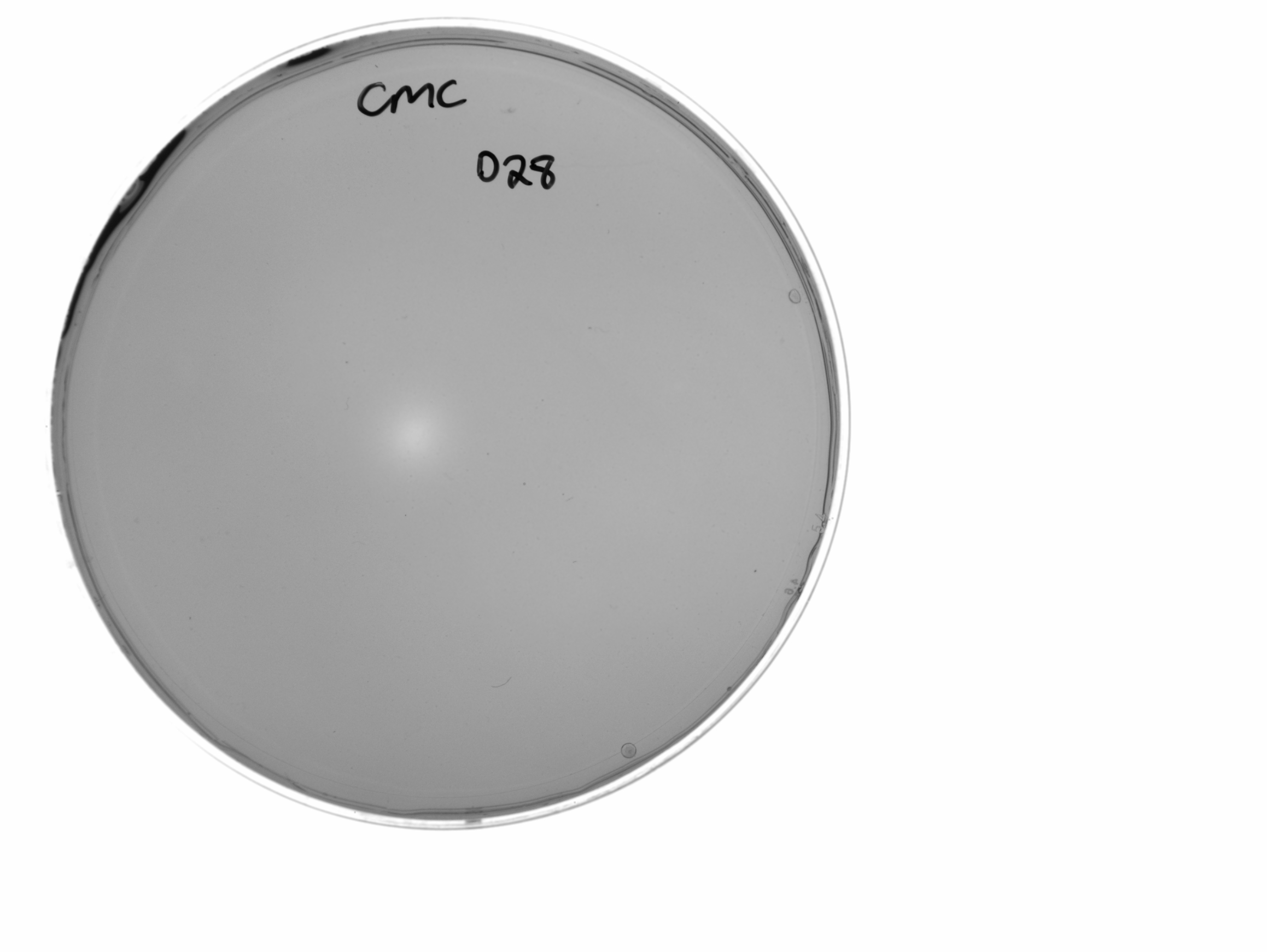 |
| *Bacillus* sp. E37 | 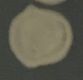 | 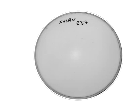 | 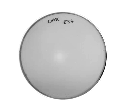 |
| *Paracoccus* sp. D32 | 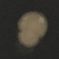 | 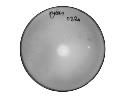 | 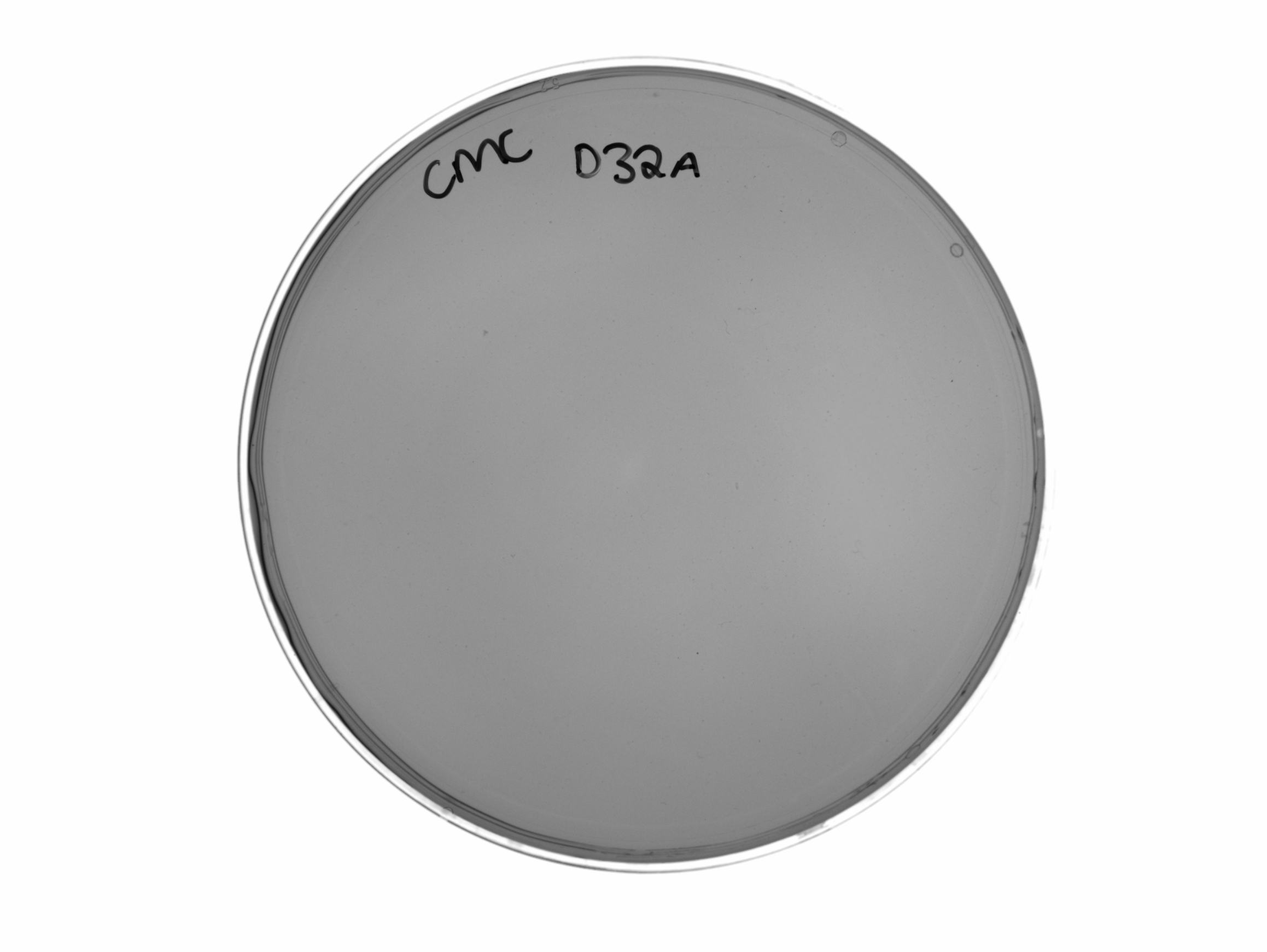 |
| *Rheinheimera* sp. D14A | 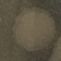 | 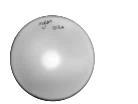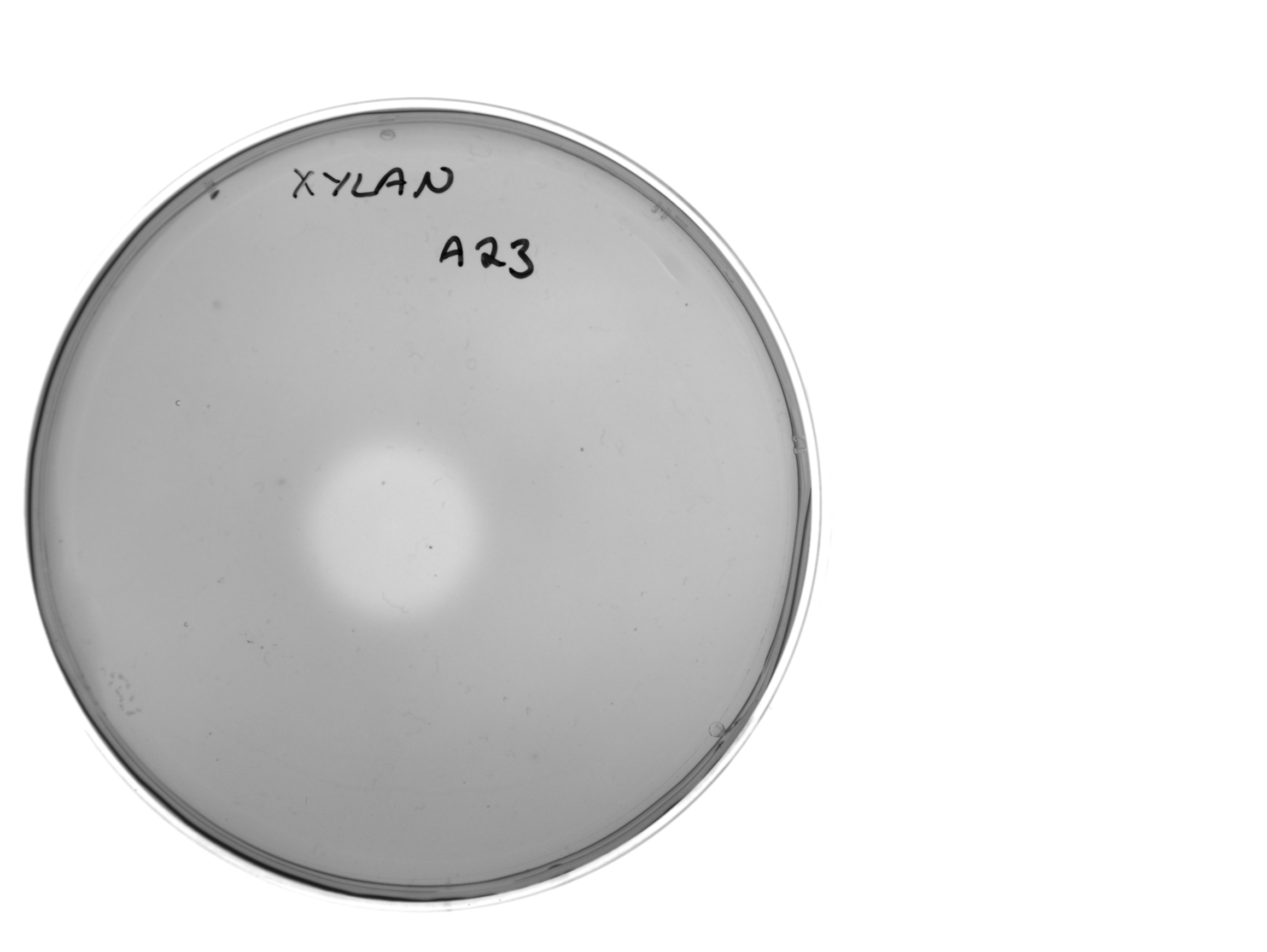 | 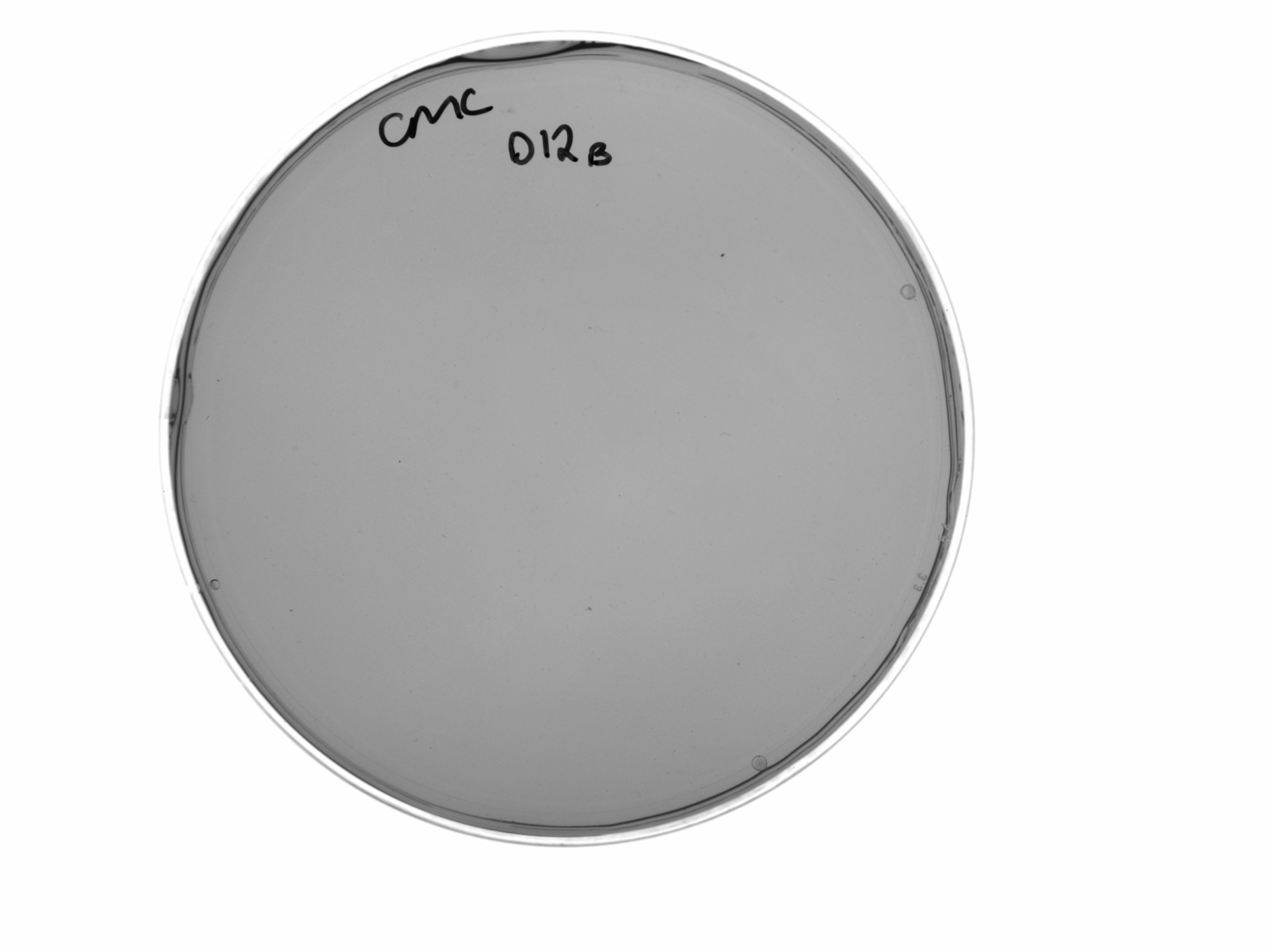 |
| *Luteimonas* sp. A23 | *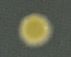* |  | 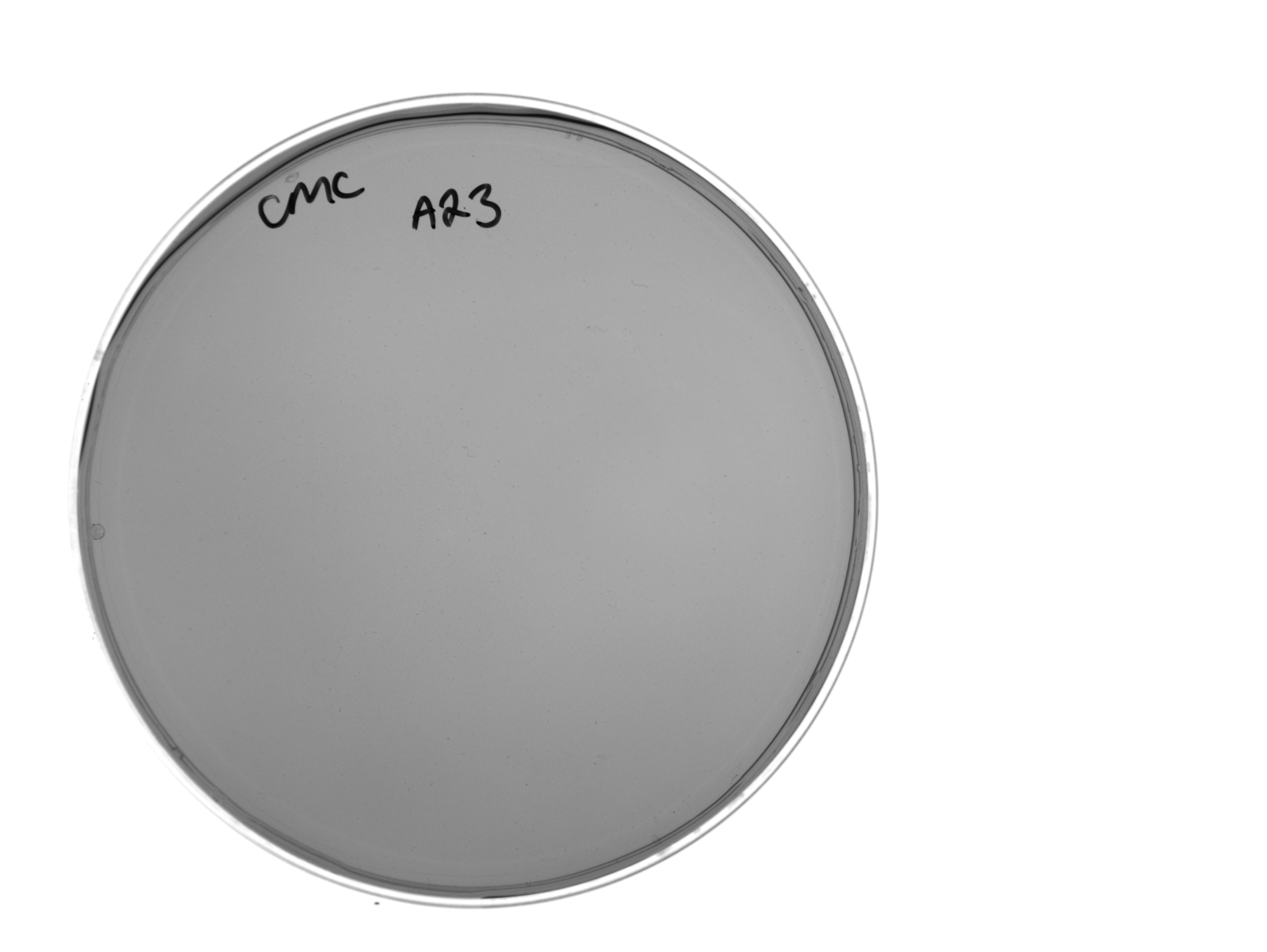 |
| *Stenotrophomonas* sp. D12B | 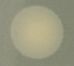 | 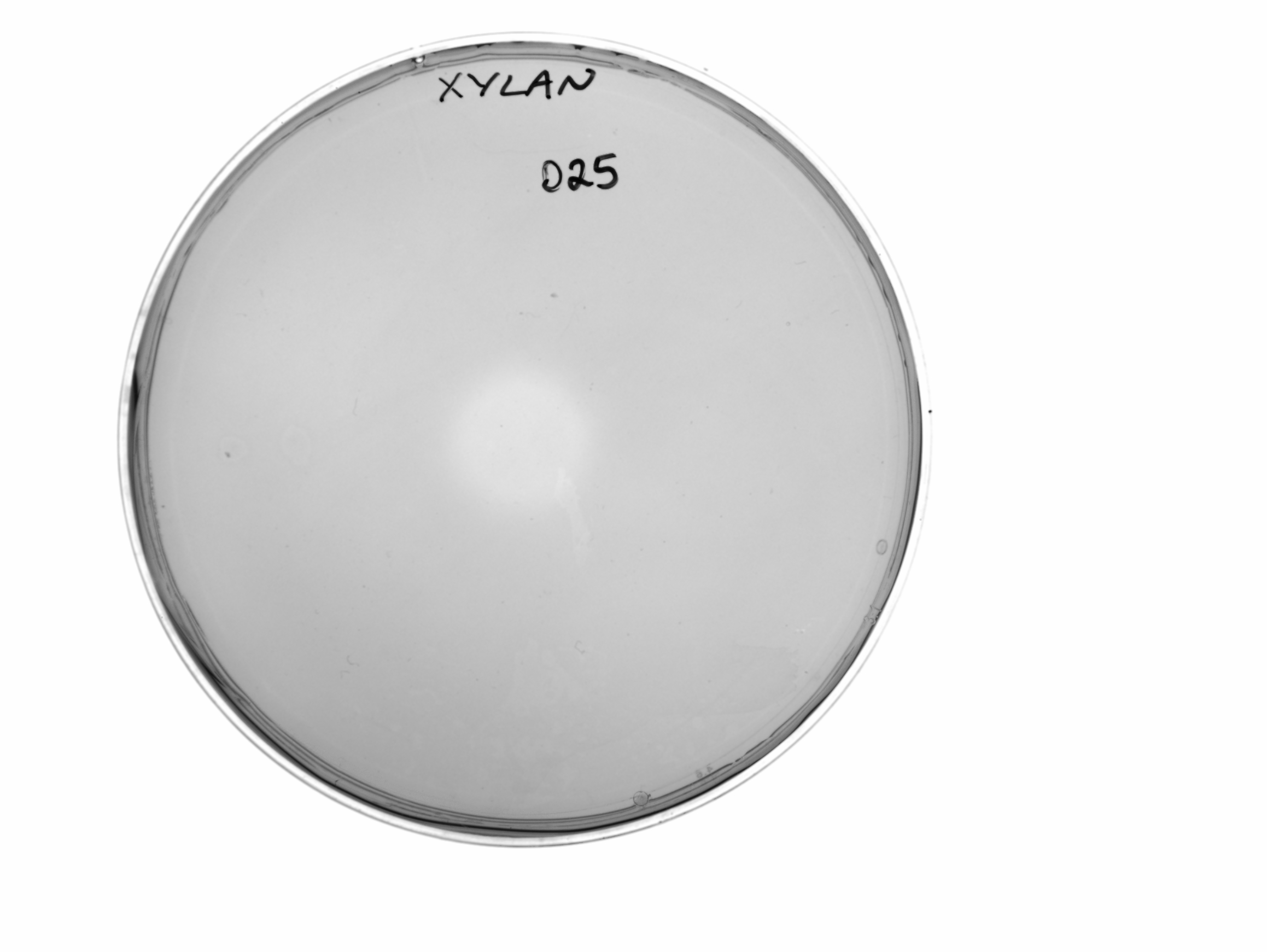 | 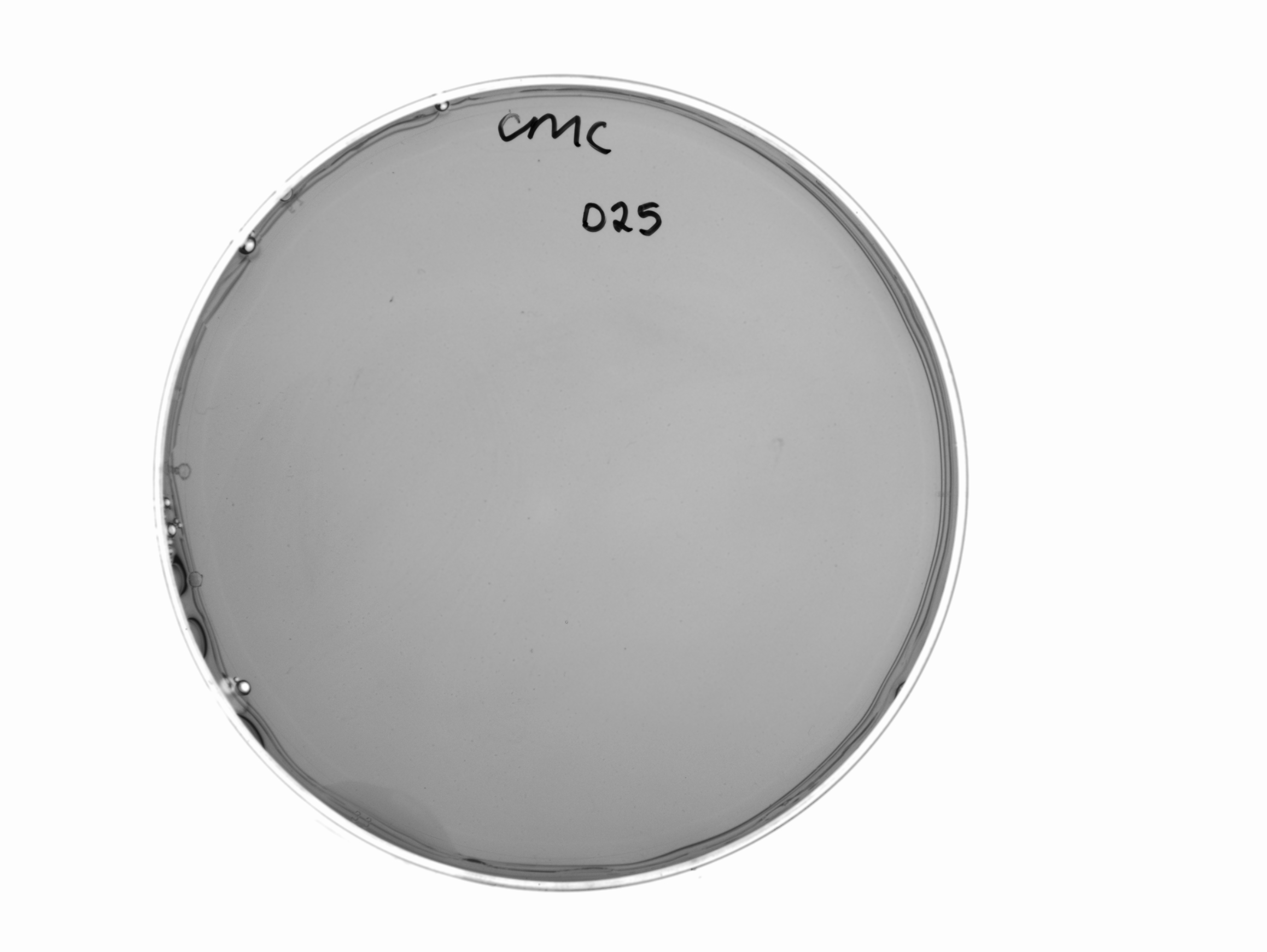 |

**Supplementary Figure S1** – Neighbour-joining phylogenetic tree based on bacterial 16S rRNA gene partial sequences. Sequences were aligned using the SILVA Incremental Aligner (SINA) and analysed by MEGA6. Isolates from this study are highlighted in bold with accession numbers provided in brackets. Bootstrap values representing percentage of 1000 replicates are shown at nodes.


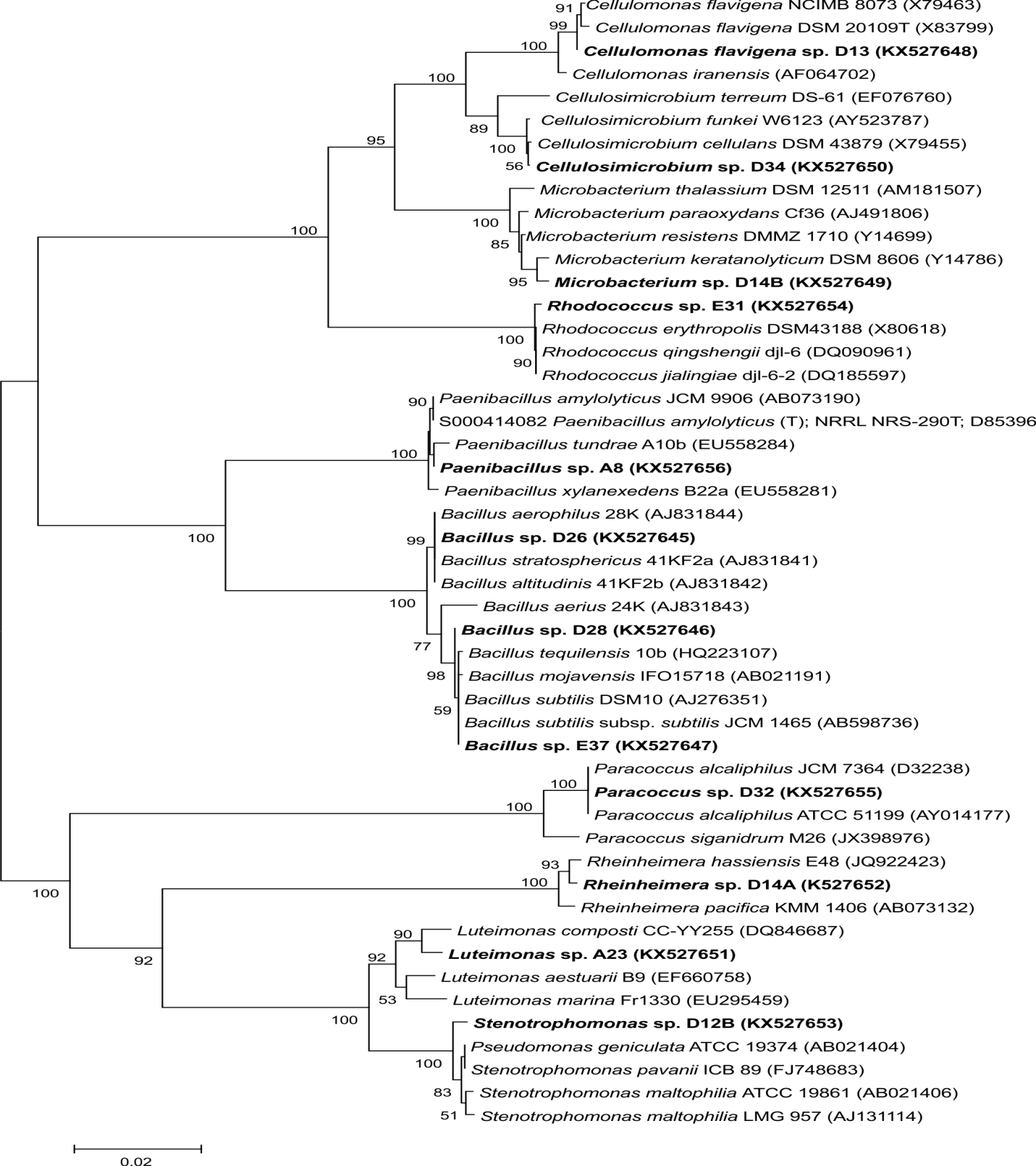


**Supplementary Figure S2** – Linear model coefficients for each species in the BEF experiment. Positive or negative coefficients indicate species contribute more or less to community productivity than an average species (Bell et al, 2009).

**Supplementary Figure S3 –** Productivity of communities on each day of the BEF experiment. Points represent mean of five replicate communities and are coloured by the presence of Paenibacillus sp. A8 (blue), C. flavigena D13 (red), both these species (green) or neither of these species (black). Productivity is the change in OD_595_ of MicroResp indicator plates after 24h.


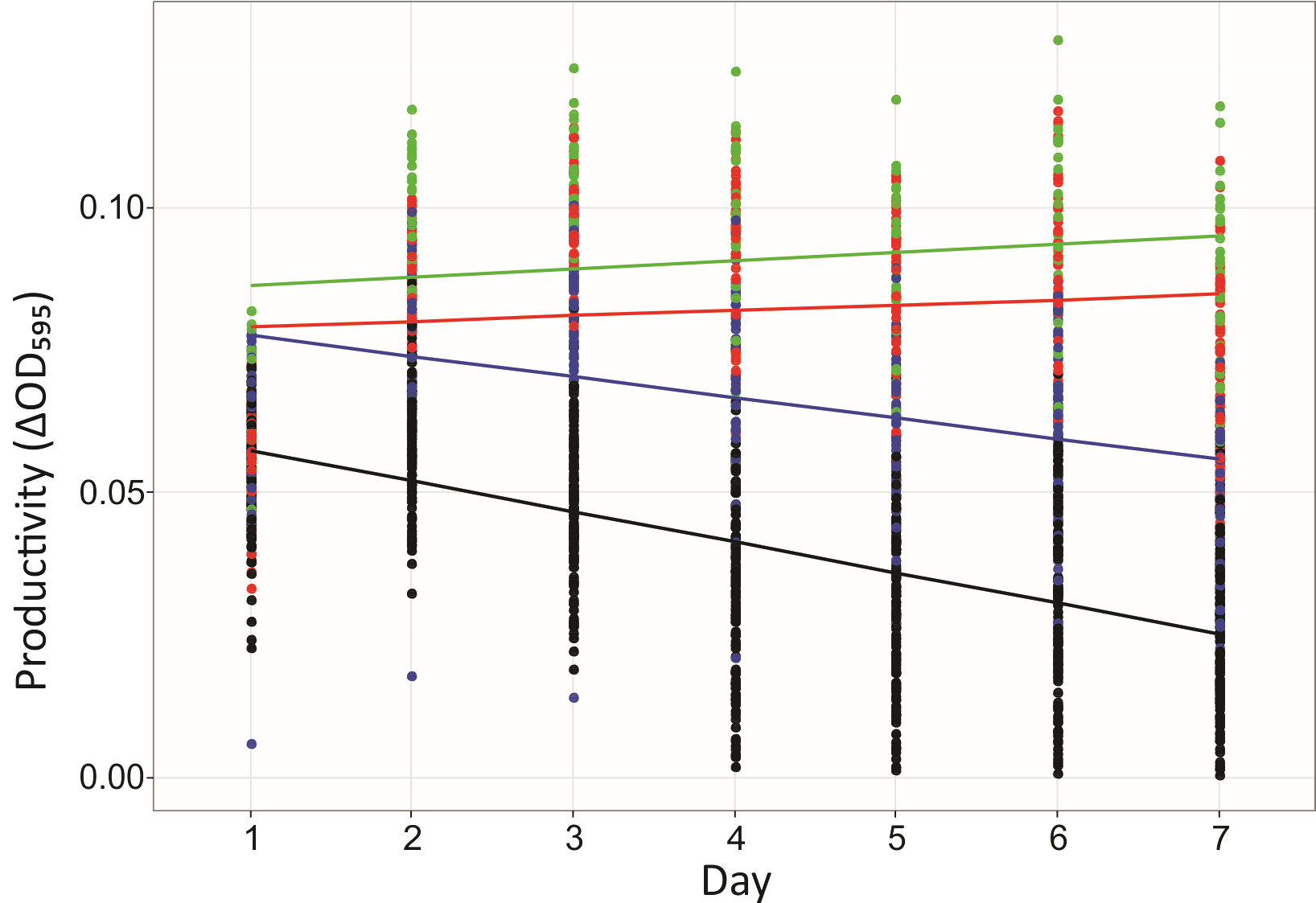


**Supplementary Table 2 –** Comparison of R^2^ and AIC values between linear models with community productivity as the dependent variable. The relationship between cumulative (sum) functional traits and maximum functional traits for each carbon source are shown. Cumulative functional traits are checked for significance when the variation explained by diversity is removed from the model to ensure these variables are not acting as a proxy of diversity.

| Explanatory variable | | R^2^ | AIC | Significance when diversity variation removed from model |
| --- | --- | --- | --- | --- |
| Species richness | | 0.19 | -266 | NA |
| Community niche | | 0.22 | -277 | NA |
| β-glucan | Sum | 0.41 | -352 | p<0.001 |
|  | Max | 0.34 | -322 | NA |
| Arabinoxylan | Sum | 0.28 | -302 | p<0.05 |
|  | Max | 0.22 | -279 | NA |
| Xylan | Sum | 0.27 | -297 | p<0.05 |
|  | Max | 0.22 | -278 | NA |
| Galactomannan | Sum | 0.18 | -266 | p>0.05 |
|  | Max | 0.15 | -255 | NA |
| Filter paper | Sum | 0.04 | -223 | p>0.05 |
|  | Max | n.s. | -212 | NA |
| Pectin | Sum | 0.20 | -272 | p>0.05 |
|  | Max | 0.07 | -232 | NA |
| Lignin | Sum | 0.18 | -264 | p>0.05 |
|  | Max | 0.02 | -219 | NA |
